# Body surface potential driven personalisation of electrophysiological digital twins in hypertrophic cardiomyopathy

**DOI:** 10.1371/journal.pcbi.1014555

**Published:** 2026-07-27

**Authors:** Shambhavi Malik, Ludovica Cicci, Abdul Qayyum, Rahul Ghelani, Ji-jian Chow, Jagdeep Singh Mohal, Zachary I. Whinnett, Amanda Varnava, Gernot Plank, Prapa Kanagaratnam, Steven A. Niederer

**Affiliations:** 1 National Heart and Lung Institute, Imperial College London, London, United Kingdom; 2 Imperial College Healthcare NHS Trust, London, United Kingdom; 3 Medical University of Graz, Graz, Austria; 4 The Alan Turing Institute, London, United Kingdom; UCI BME: University of California Irvine Department of Biomedical Engineering, UNITED STATES OF AMERICA

## Abstract

**Background:**

Hypertrophic cardiomyopathy (HCM) is associated with marked inter-patient heterogeneity in ventricular electrophysiology, contributing to arrhythmic risk that is insufficiently captured by current clinical methods. Electrocardiographic imaging (ECGI) provides high-density body surface potential (BSP) measurements but remains largely descriptive. Computational modelling offers a mechanistic framework to interpret BSP signals in terms of underlying tissue-level properties.

**Methods and findings:**

We developed a BSP-driven workflow to construct patient-specific electrophysiology (EP) models of HCM by integrating multimodal clinical imaging with Bayesian model calibration. Anatomically detailed torso-heart finite-element models were generated for 17 HCM patients using thoracic computed tomography (CT), cardiac magnetic resonance imaging (CMR), and 252-electrode BSP recordings. Ventricular depolarisation and repolarisation were simulated using a reaction-eikonal (RE) formulation coupled to a biophysically detailed ToR-ORd-dynCl ionic model. Emulator-based Bayesian history matching (HM) was used to personalise EP parameters, with staged calibration of QRS and T-wave morphology informed by targeted sensitivity analysis. The calibrated cohort reproduced clinical BSP morphology with Pearson correlation coefficient (PCC) ≥0.6 for a median of 94.0% (IQR: 91.6 to 96.8%) of electrodes, achieving a median PCC of 0.89 (IQR: 0.80 to 0.94) across the full 252-electrode vest. Calibration substantially reduced uncertainty in the high-dimensional EP parameter space while yielding physiologically plausible conduction and repolarisation properties. Models calibrated exclusively to sinus rhythm robustly generalised to right-ventricular (RV) apical pacing without parameter retuning, reproducing clinically observed pacing-induced trends in depolarisation and repolarisation. Exploratory analysis revealed biologically consistent associations between inferred EP parameters and patient demographics.

**Conclusions:**

This study demonstrates that high-density BSP data can be used to functionally personalise mechanistic EP in HCM. The framework captures intrinsic patient-specific EP properties and generalises beyond the calibration condition, supporting its use for mechanistic investigation of arrhythmogenic substrate.

## Author summary

HCM shows significant variability in how electrical signals propagate through the heart, contributing to differences in arrhythmia risk between patients. While BSPs can non-invasively measure cardiac electrical activity, these signals are difficult to interpret in terms of the underlying myocardial properties. We developed a computational framework that combines BSP signals with patient-specific anatomical models to create personalised EP digital twins. We used high-resolution medical imaging and Bayesian calibration techniques to adjust model parameters and reproduce clinically measured BSPs. We tested whether models calibrated to sinus rhythm could generalise to a different electrical activation pattern by simulating RV pacing without further tuning. Calibrated models were able to reproduce patient-specific BSP signals with good accuracy and captured key electrical changes observed during pacing. The inferred model parameters were physiologically plausible and showed consistent relationships with patient characteristics such as age and body size. These findings show that BSP-driven digital twins can capture intrinsic electrical properties of individual HCM hearts rather than simply fitting surface signals. This approach provides a foundation for mechanistic studies of arrhythmogenic substrate and future in silico investigations of disease progression and therapy.

## Introduction

Hypertrophic cardiomyopathy (HCM) is the most common genetic cardiac disorder, with an incidence of 1 in 500 individuals worldwide [1]. It is predominantly caused by pathogenic variants in genes encoding sarcomeric proteins, leading to abnormal myocardial architecture and impaired contractile function [2,3]. Approximately 40–60% of cases follow an autosomal dominant inheritance pattern [4], resulting in substantial familial clustering and lifelong disease burden. Clinically, HCM is characterised by left ventricular (LV) hypertrophy, often asymmetric, associated with diastolic dysfunction, myocardial fibrosis, and, in some patients, LV outflow tract obstruction (LVOTO) [5]. Disease expression is highly heterogeneous. While many individuals remain asymptomatic or mildly symptomatic, others develop serious complications including ventricular arrhythmias, heart failure, stroke, or sudden cardiac death (SCD) [6]. This marked inter-patient variability presents persistent challenges for clinical assessment and management, motivating the need for approaches that better capture patient-specific disease mechanisms [7].

Ventricular arrhythmias represent a major source of morbidity in HCM and underlie its most severe clinical outcomes. These arrhythmias arise from a complex electrophysiological substrate shaped by myocardial disarray, interstitial fibrosis, and spatially heterogeneous conduction and repolarisation properties [8,9]. Although current risk stratification frameworks incorporate demographic, structural, and clinical history variables, they do not directly characterise the underlying electrical abnormalities responsible for arrhythmogenic behaviour [10]. As a result, approaches that directly quantify patient-specific electrical properties are required to advance mechanistic understanding and improve risk assessment in HCM.

Electrophysiological studies have demonstrated that HCM is associated with pronounced spatial heterogeneity in ventricular activation and repolarisation, including regional conduction delay and increased spatial heterogeneity of repolarisation gradients, that are not adequately captured by the standard 12-lead electrocardiogram (ECG) [11]. Owing to its limited spatial sampling, the conventional ECG provides insufficient resolution to characterise these regional abnormalities [12]. Electrocardiographic imaging (ECGI) addresses this limitation by combining high-density body surface potential (BSP) recordings with patient-specific torso-heart geometry derived from medical imaging to reconstruct epicardial electrical activity with high spatial resolution [12,13]. In HCM, ECGI has revealed extensive electrical heterogeneity even in patients with normal 12-lead ECGs, underscoring its sensitivity to functional abnormalities that extend beyond overt structural disease [11]. The richness of this modality derives from the underlying high-density BSPs and establishes them as a powerful non-invasive tool for characterising the electrical phenotype of HCM.

Despite the spatial richness of BSPs and the descriptive power of ECGI reconstructions, neither directly links the observed electrical patterns to the tissue properties and mechanisms that produce them. Patient-specific computational modelling of cardiac electrophysiology (EP) offers a complementary mechanistic framework by enabling explicit representation of cardiac structure and electrical function within a unified, physics-based model [14]. Over the past two decades, cardiac digital twins have progressed from generic models toward increasingly personalised representations incorporating patient-specific anatomy and pathology [15]. In EP, such models have demonstrated utility for mechanistic investigation and procedural planning, including applications in ventricular tachycardia ablation and atrial fibrillation treatment [16–18]. In HCM, recent studies integrating fibrosis distributions from late gadolinium enhancement (LGE) scans further highlight the potential of patient-specific modelling to investigate arrhythmogenic substrate [19].

Integrating high-density BSPs directly with computational modelling and statistical calibration provides a complementary pathway toward functional personalisation of cardiac digital twins. In this study, we present a comprehensive workflow for constructing electrophysiological digital twins of HCM patients by integrating multimodal clinical imaging with forward modelling and Bayesian calibration. Thoracic computed tomography (CT), cardiac magnetic resonance imaging (CMR), and 252-electrode BSP recordings are combined with emulator-based Bayesian history matching (HM) to calibrate a reaction-eikonal (RE) modelling framework. This approach enables simulation of patient-specific conduction properties, His-Purkinje activation patterns, and transmural repolarisation gradients to reproduce BSPs during sinus rhythm. To assess physiological validity beyond the calibration domain, model generalisation is evaluated by simulating right ventricular (RV) pacing without parameter retuning. This work establishes a validated framework for BSP-driven functional personalisation of HCM digital twins and provides a mechanistic foundation for future studies of patient-specific EP in this heterogeneous disease.

## Materials and methods

### Ethics statement

This study complied with the Declaration of Helsinki and the protocol was approved by the Fulham Research Ethics Committee (Health Research Authority, references 14/LO/1318 and 17/LO/1660). The study was conducted in accordance with the local legislation and institutional requirements. Each patient provided written informed consent, and images were anonymised prior to analysis.

### Overview

**Fig 1:**
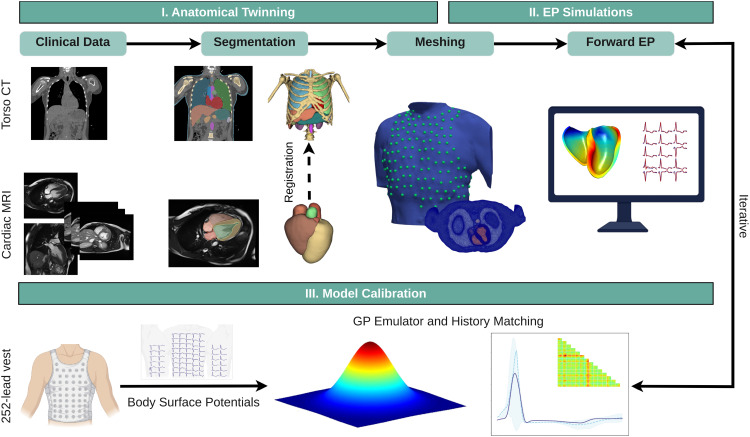
Workflow for developing EP digital twins. **(I)** Multimodal clinical data, including thoracic CT and CMR are segmented and co-registered to construct patient-specific finite-element meshes. **(II)** Ventricular EP and BSPs are simulated using a RE framework. **(III)** Model parameters are iteratively calibrated against clinically recorded BSPs using emulator-based HM, yielding electrophysiological digital twins. The proposed pipeline is an extension of our previous work [20].

We developed EP digital twins of HCM to reproduce clinically recorded BSPs, expanding on our previous work [20]. Multimodal clinical imaging was used to construct anatomically detailed torso-heart models, which were discretised using finite-element meshes and simulated within a RE framework. Model personalisation was achieved using emulator-based HM to calibrate patient-specific model parameters. Fig 1 depicts the workflow overview.

### Study cohort

The dataset used comprised 17 patients with HCM drawn from a larger prospective cohort recruited at Hammersmith Hospital, Imperial College Healthcare NHS Trust. HCM diagnosis followed European Society of Cardiology (ESC) guidelines [21], and patients with secondary causes of hypertrophy were excluded. Complete patient characteristics are detailed in Chow et al. (2021) [22].

Participants underwent a standardised multimodal imaging protocol comprising BSP mapping, thoracic CT, and CMR imaging. BSP signals were acquired using a 252-electrode CardioInsight v3.1 ECGI vest (Medtronic, Minneapolis, MN, USA) during a Bruce Protocol treadmill exercise test, followed by ten minutes of supine recovery. Non-contrast thoracic CT was performed on a Siemens SOMATOM Definition AS scanner to provide torso geometry for the forward model and BSP acquisition setup. High-resolution CMR included long and short-axis cine sequences. Recorded continuous clinical demographics included age, body mass index (BMI), body surface area (BSA), ESC SCD risk score, maximal LV wall thickness, left atrial diameter, and LVOTO gradient. Categorical variables included sex, history of non-sustained ventricular tachycardia (NSVT), and history of syncope.

### Anatomical twin generation

Patient-specific torso-heart geometries were constructed by segmenting and co-registering multimodal medical imaging data [20]. Abdominal and thoracic cavities were segmented from non-contrast thoracic CT. Owing to the limited soft-tissue resolution of non-contrast CT, whole-heart anatomy was segmented from CMR. To integrate high-fidelity cardiac anatomy within the CT-derived torso, CMR-based whole-heart segmentations were rigidly registered to the CT geometry, as shown in S1 Fig. Workflow steps are detailed in S1 Appendix.

**Fig 2:**
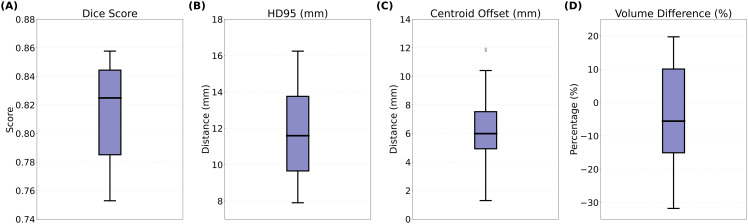
Registration validation metrics across the cohort. Box plots show (A) Dice similarity coefficient, (B) HD95 (mm), (C) centroid offset (mm), and (D) volume difference (%). Boxes represent the interquartile range (IQR), horizontal lines indicate median values, and whiskers extend to 1.5 × IQR.

Registration accuracy was assessed using both qualitative and quantitative criteria. Qualitative validation involved visual inspection of the registered CMR heart overlaid within the CT torso, ensuring anatomically consistent positioning relative to surrounding thoracic structures, including the lungs, great vessels, and chest wall. Quantitative evaluation compared the registered CMR heart against the CT-derived heart mask using Dice similarity coefficient, centroid offset, volume difference, and the 95th percentile Hausdorff distance (HD95). Across the cohort, registration achieved median values of: Dice score 0.825 (IQR: 0.785 to 0.845), HD95 11.6 mm (IQR: 9.6 to 13.5 mm), centroid offset 6.0 mm (IQR: 5.0 to 7.5 mm), and volume difference -5.6% (IQR: -15.0 to 10.0%) (Fig 2).

Volumetric finite-element meshes were generated from the registered torso-heart segmentations using Simpleware (Synopsys, Mountain View, CA, USA). Meshes were unstructured, conformal, and boundary-fitted, with a target edge length of 800±100 μm in the LV/RV myocardium and 2±1 mm in all non-myocardial tissues. The resulting meshes comprised 23 distinct tissue compartments, as listed in S1 Table.

Universal Ventricular Coordinates (UVCs) [23] were computed to provide an intrinsic parametrisation of ventricular anatomy, by defining apico-basal (*z*), transmural (ρ), rotational (ϕ), and chamber-wise (ν) coordinates. As patient-specific diffusion tensor imaging data was not available, biventricular myocardial fibre orientations were assigned using the Laplace-Dirichlet rule-based method [24]. This required endocardial and epicardial fibre and sheet angles as reported in S2 Table. UVC generation and fibre assignment were automated using Meshtool [25] and Cardiac Arrhythmia Research Package (CARPentry) [26].

The 252 ECGI vest electrodes were mapped onto the torso mesh by first scaling the electrode point cloud to the mesh space in Paraview (v5.11.2), followed by closest point projection to snap electrodes onto the mesh surface. The positional displacement introduced by this projection is bounded by the local mesh element size (2±1 mm). A reference electrode was selected manually at an inferior-anterior left-sided location on the thorax, approximating a left-leg reference for torso potential recordings. The final electrode configuration is shown in S2 Fig.

### Electrophysiology simulation setup

Bi-ventricular EP was modelled based on the RE Lead Field formulation [20,27], with full methodological details provided in S1 Appendix. Ventricular depolarisation during sinus rhythm was mediated by a simplified fascicular-based representation of the His-Purkinje system (HPS) (S3B Fig). To capture the functional effect of Purkinje-myocyte coupling, a one-element-thick (average thickness of 600 μm) subendocardial (SE) layer was defined (S3A Fig), extending to a prescribed percentage of the ventricular wall in the apico-basal direction. While tissue patches within the fascicles were assumed to depolarise instantaneously, the SE layer enabled rapid intramural spread following activation. Fascicle UVC locations and corresponding activation timings are provided in S3A and S3B Table.

Myocardial conduction velocity (CV) was modelled as transversely isotropic, with preferential propagation along the fibre direction. Spatial heterogeneity was prescribed independently for the LV and RV through apico-basal and transmural gradients, as described in S1 Appendix, consistent with experimental measurements and prior computational studies [27–29]. The SE layer was assigned a higher CV relative to the bulk myocardium, with a fixed anisotropy ratio. All CV parameters and gradients are summarised in S4 Table.

Ventricular repolarisation dynamics were governed by both the activation sequence and intrinsic regional heterogeneity in cellular EP. Cellular EP was represented using the ToR-ORd-dynCl ionic model [30], with HCM-specific ionic remodelling incorporated following Tomek et al. [31], to reproduce action potential morphologies and durations characteristic of HCM (S5 Table). Ion-channel conductance gradients were imposed separately in the LV and RV along both apico-basal and transmural directions, as listed in S6 Table. Single-cell ionic parameters are provided in S7 Table, and the assignment of spatial heterogeneities is detailed in S1 Appendix.

A lead-field-based approach [32] was used to recover BSPs at ECGI electrode positions. Computation of lead fields and subsequent signals required modelling passive conduction through the torso. All non-myocardial tissues and the bath were considered homogeneous isotropic conductive media with conductivity values listed in S8 Table. Myocardial conductivities were mapped from CV values using a lookup table approach, detailed in S1 Appendix. Overall, the EP framework comprised 122 parameters and simulations were performed using CARPentry [26].

### Model calibration

To personalise EP digital twins, we calibrated model parameters against clinically recorded BSPs using a multi-stage framework. Given the high dimensionality of both BSP time series (252 electrodes × 500 ms) and the parameter space (122 parameters), the calibration approach comprised three components:

1. dimensionality reduction of simulated and clinical signals,
2. identification of dominant parameters influencing depolarisation and repolarisation dynamics, and,
3. iterative constraint of the plausible parameter space using emulator-based HM.

Owing to the distinct physiological mechanisms governing QRS-complex and T-wave morphology, depolarisation and repolarisation were calibrated separately. Model fidelity was evaluated by comparing simulated and clinical BSP morphology.

#### Signal representation and dimensionality reduction.

Clinical BSPs were recorded as 10-beat time-series for all 252 ECGI vest electrodes. In contrast, the forward EP model simulated single-beat waveforms. To enable consistent comparison, individual beats were extracted from clinical recordings as detailed in S1 Appendix. Signal dimensionality was reduced using a two-stage strategy of electrode-level time-series clustering followed by projection onto a low-dimensional principal component (PC) basis.

First, redundancy in clinical recordings was reduced by clustering signals from all 252 vest electrodes based on waveform similarity using the TimeSeriesKMeans [33] algorithm with Euclidean distance metric. For each cluster, the electrode with signal closest to the centroid was selected as representative.

Cluster count (*k*) was selected using leave-one-out cross-validation (LOOCV) on each patient’s BSP recordings. For each candidate *k* ∈ {10, 20, ..., 100}, every electrode was held out in turn, while TimeSeriesKMeans was fit to the remaining electrode signals. The held-out signal was then assigned to its nearest cluster centroid, and the resulting Euclidean reconstruction error was recorded.

Reconstruction-error curves were averaged across folds within each patient, and the cohort-median curve was computed. The elbow of both per-patient curves and the cohort-median curve was identified using the kneedle algorithm [34]. To ensure both data-driven sufficiency and consistent spatial coverage across patients, *k* = 50 was selected as the value that lies at or above the elbow of every individual patient’s curve.

Principal component analysis (PCA) was then applied to construct a compact representation of signal morphology. PCA projects high-dimensional time-series data onto an orthogonal basis capturing dominant modes of variation, enabling each signal to be expressed as a linear combination of PCs with corresponding PC scores. For each patient, PCA was performed on simulated BSPs generated by sampling the EP model parameter space using a Latin hypercube design (N = 750 samples). Signals from the sampled electrodes were extracted over a single cardiac cycle and concatenated into a spatio-temporal vector of dimension $N_{elec}\times N_{t}$ where $N_{elec}=50$ electrodes and $N_{t}$ denotes the number of time samples. PCA mapped this concatenated signal onto a reduced $N_{PCA}$ dimensional basis, with $N_{PCA}\ll N_{elec}\times N_{t}$.

The number of retained PCA modes was selected to explain 95% of total signal variance. Leading modes captured dominant features, while higher-order modes represented subtle variations in shape and timing. Both clinical and simulated BSPs were projected onto this patient-specific basis. The resulting PCs formed the feature space for HM, with calibration carried out entirely in the reduced PC space. Waveform-level comparisons against clinical recordings were performed as a separate post-calibration step, without inverse PCA reconstruction.

#### Sensitivity analysis and parameter selection.

Sensitivity analysis (SA) was performed to identify model parameters that exert dominant influence on BSP morphology and to reduce calibration complexity. The model initially included 122 uncertain parameters, making global SA (GSA) of the full 3D EP model computationally prohibitive. A multi-stage strategy was therefore adopted, focusing separately on ventricular depolarisation and repolarisation.

At the single-cell level, variance-based GSA was performed for the ToR-ORd-dynCl ionic model, computing sensitivity indices for scalar cardiac action potential features. A Saltelli sampling scheme [35] [36] with 1024 base samples was used to explore the ionic parameter space. To enable efficient estimation of Sobol’ total-effect indices, Gaussian process emulators (GPEs) were trained on single-cell simulation outputs, allowing sensitivity indices to be computed while accounting for emulator uncertainty. Ionic parameters were ranked according to their maximum total-effect Sobol’ index across all outputs. Those explaining 90% of the cumulative output variance were retained (S4 Fig), with remaining inputs fixed at baseline values as specified in S7 Table.

At the tissue and organ scale, screening was performed using one-at-a-time (OAT) SA. Each parameter was perturbed individually to its prescribed upper and lower bounds, keeping all others fixed at baseline. Sensitivities were quantified using normalised linear regression coefficients (detailed in S1 Appendix) computed from changes in simulated BSP projected onto the pre-computed PCA basis. Parameters with negligible first-order effects were assumed to have negligible higher-order effects. The cumulative variance for each parameter across all PCs was used to quantify its influence. Those explaining 90% of waveform variance were retained for calibration, with the rest held at baseline (see Supplementary Tables). Because the QRS complex and T-wave reflect distinct physiological mechanisms, OAT screening was applied separately to each signal component, enabling focused parameter refinement while maintaining model fidelity (S5 and S6 Figs).

#### Emulator-based history matching.

Parameters identified as influential through SA were calibrated using an emulator-based HM framework, implemented using the GPErks emulation tool (https://github.com/stelong/GPErks) [37]. The framework involved an iterative process of training GPEs to efficiently explore the input parameter space, followed by ruling out implausible combinations using Bayesian HM, as depicted in S7 Fig.

A separate emulator was trained for each of the $N_{PCA}$ retained PCs, providing both mean estimates and associated uncertainty quantifying approximation errors. Training datasets were generated by sampling the parameter space and running forward EP simulations for each sample. Full training details are provided in S1 Appendix. HM constrained the input space to regions consistent with observed data by iteratively ruling out implausible parameter combinations, as described in S1 Appendix. This strategy is well suited to complex EP models with high-dimensional, uncertain inputs and computationally expensive simulations. The entire GPE-based HM pipeline was implemented in the reduced PC space.

**Fig 3:**
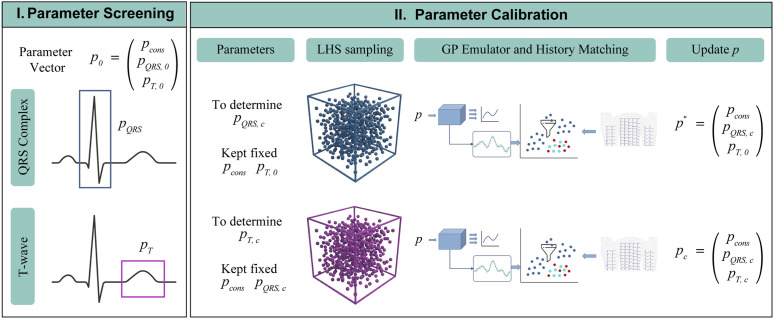
Two-stage parameter screening and calibration strategy for ventricular depolarisation and repolarisation. Model parameters are partitioned into fixed parameters ($p_{cons}$), depolarisation-related parameters ($p_{QRS}$), and repolarisation-related parameters ($p_{T}$). **(I)** Initial parameter screening identifies subsets of influential parameter subsets for each phase of the cardiac cycle. **(II)** Calibration is performed sequentially: first calibrating $p_{QRS}$ keeping $p_{T}$ fixed to their baseline values ($p_{T,0}$), then calibrating $p_{T}$ while $p_{QRS}$ are fixed to their calibrated values ($p_{QRS,c}$). Gaussian process emulators and Bayesian history matching iteratively rule out implausible regions at each stage, yielding patient-specific parameter sets.

HM was applied in two stages: parameters governing ventricular depolarisation were first calibrated against QRS morphology, followed by calibration of repolarisation parameters against T-wave morphology. This staged approach (Fig 3) reduced parameter interactions, improved emulator performance, and enabled targeted model refinement for each phase of the cardiac cycle.

The final output of the HM framework was a patient-specific not-ruled-out-yet (NROY) ensemble of plausible parameter combinations that produced simulated BSPs consistent with observed clinical signal morphology. Representative best-fit parameter set from this ensemble was retained for subsequent validation against clinical data.

### Model assessment

Model performance was evaluated using a two-stage validation strategy designed to assess both fidelity to observed clinical signals and robustness under altered activation patterns. Internal validation quantified agreement between simulated and clinical BSPs under sinus rhythm, while pacing validation tested model generalization to an unseen altered activation protocol without additional parameter tuning.

#### Internal validation.

To assess fidelity of the calibrated EP model, simulated and clinical BSPs were compared, first on the reduced electrode subset used during calibration, then on the full 252 electrode configuration. Evaluation focused on waveform morphology and temporal alignment rather than explicit parameter recovery.

Raw clinical signals were pre-processed using the NeuroKit2 toolkit [38] to remove baseline wander and powerline interference, as detailed in S1 Appendix. Both simulated and clinical BSPs were normalised using z-score normalisation to remove amplitude bias and enable morphology-based comparison. To ensure consistent temporal alignment, signals were aligned using the first QRS peak as a fiducial landmark. Initially, QRS peak was detected in the simulated signal within a 140 ms search window from the QRS-onset. The corresponding peak in the clinical signal was then identified in the same window, constrained to match the polarity of the simulated peak. Signals were aligned by shifting one relative to the other so that the two peaks coincided, with edge-padding to preserve signal length without clipping.

Waveform similarity was then quantified at each electrode using the Pearson correlation coefficient (*PCC*), defined as,


$$\begin{array}{c} PCC=\frac{\sum_{i=1}^{n}(x_{i}-\bar{x})(y_{i}-\bar{y})}{\sqrt{\sum_{i=1}^{n}(x_{i}-\bar{x}{)}^{2}\sum_{i=1}^{n}(y_{i}-\bar{y}{)}^{2}}} \end{array}$$

where $x_{i}$ and $y_{i}$ denote the simulated and clinical BSP samples at time point *i*, $\bar{x}$ and $\bar{y}$ their respective means and *n* the number of time points. *PCC* values range from -1–1, with values closer to 1 indicating stronger morphological agreement.

For each parameter combination in the final NROY ensemble, *PCC* values were computed at all 50 sampled locations. Calibration quality was assessed by the number of channels achieving PCC≥0.6 (moderate-to-good agreement) and PCC≥0.8 (good agreement), based on established literature [39,40]. The parameter set maximizing the number of electrodes with PCC≥0.6 was selected as the best-fit configuration.

The best-fit was then used to perform a full-forward EP simulation generating BSPs at all 252 vest electrode locations. Electrodes with missing recordings or with traces lacking identifiable QRS-T morphology were excluded. Model assessment was repeated using the same signal processing, normalisation, alignment, and *PCC*-based comparison pipeline. Crucially, no additional parameter tuning was performed at this stage, ensuring that the full-electrode evaluation provided an independent test of model consistency beyond the calibration subset.

#### Pacing validation.

To evaluate whether models calibrated exclusively to sinus rhythm generalized to unseen activation patterns, RV apical pacing was simulated without any additional parameter calibration. As paced BSP recordings were not available for the calibrated cohort, model generalization was assessed using a trend-based validation strategy. An independent, unseen, external HCM cohort (*n* = 13) with paired sinus rhythm and RV-paced recordings was used (EMORI-HCM patient database, J.S. Mohal and Z.I. Whinnett, National Heart and Lung Institute, Imperial College London).

For each patient in the EMORI-HCM dataset, pre-computed depolarisation and repolarisation metrics tabulated per precordial lead (V1–V8) under both sinus rhythm and RV pacing were provided. These ECG-derived metrics are defined in S9 Table. From this available set, metrics were retained for validation based on the magnitude, consistency, and statistical significance of their pacing-induced changes (Δ = pacing - sinus), assessed using paired Wilcoxon signed-rank testing in the external cohort.

For each calibrated digital twin, RV apical pacing was simulated using the best-fit parameter set identified from sinus rhythm calibration. BSPs were recomputed and pacing-induced changes in the retained ECG metrics were quantified relative to the corresponding sinus rhythm simulations. As ECGI vest BSPs do not provide standard 12-lead ECGs, lead-specific metrics were derived from anatomically corresponding vest electrodes. Agreement was evaluated based on the directionality, relative magnitude, and spatial consistency of pacing-induced metric changes between the calibrated digital twins and the external clinical cohort.

## Results

EP models were successfully calibrated to BSPs for 17 HCM patients using a two-stage HM framework. Models achieved moderate-to-good morphological agreement with clinical signals, and performance remained consistent from 50 representative electrodes to the full 252-electrode vest. Moreover, calibrated models reproduced key pacing-induced electrophysiological changes without additional parameter tuning, demonstrating physiological validity beyond the calibration domain. Further analysis revealed 30 nominal associations between calibrated parameters and demographic or clinical phenotypes that require further exploration in larger cohorts. All subsequent values are reported as median (IQR: Q1 to Q3) unless otherwise stated.

### Patient-specific model calibration

**Fig 4:**
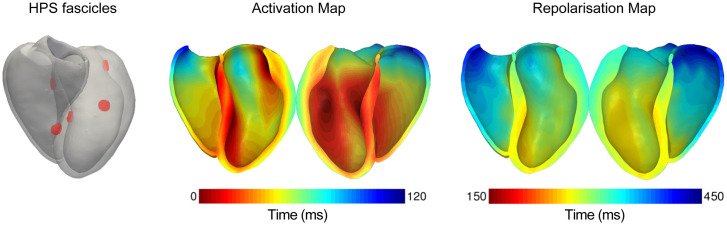
Representative activation and repolarisation maps. (Left) Patient-specific HPS configuration, with red markers indicating early activation sites. (Centre) Activation map ranging from 0 to 120 ms. (Right) Repolarisation map ranging from 150 to 450 ms.

Sequential calibration using GPE-based HM converged efficiently, requiring a median of 6 total iterations (IQR: 5–9). This included 2 iterations (IQR: 1–3) for QRS-complex calibration and 4 iterations (IQR: 3–5) for T-wave calibration (S8 Fig). For each patient, the parameter set from the final NROY ensemble that maximized electrode-wise correlation with clinical BSPs was selected as the best-fit configuration. Fig 4 shows activation and repolarisation maps for a calibrated patient with corresponding calibrated locations of HPS fascicles.

**Fig 5:**
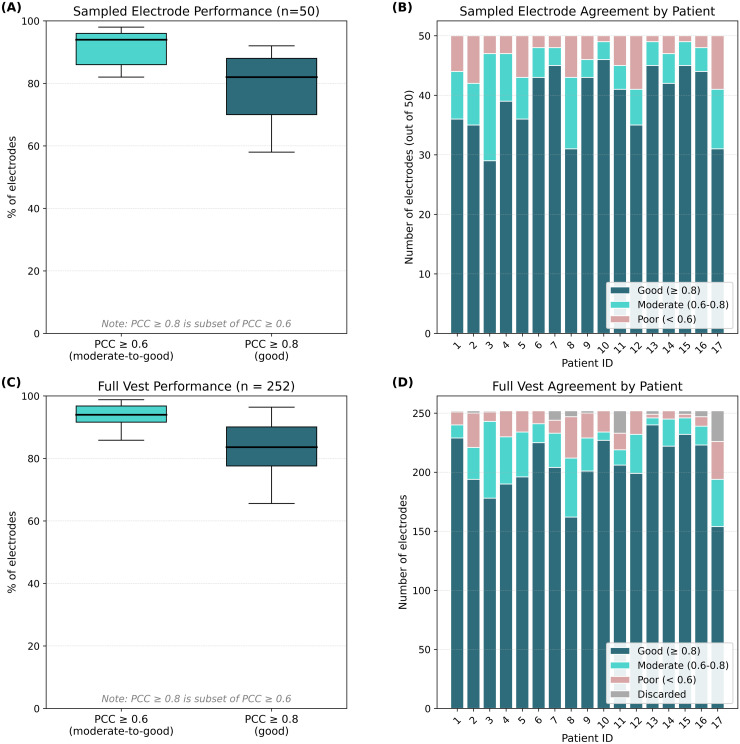
Calibration performance on representative and full vest electrode sets. **(A)** Distribution across the cohort of the percentage of 50 representative electrodes achieving moderate-to-good (PCC≥0.6, left) and good (PCC≥0.8, right) morphological agreement between simulated and clinical BSPs. **(B)** Per-patient breakdown of agreement across representative electrodes, partitioned into good (PCC≥0.8), moderate (0.8>PCC≥0.6), and poor (*PCC* < 0.6) categories. **(C)** Corresponding cohort-level performance across all 252 ECGI vest electrodes using the same agreement thresholds. **(D)** Per-patient agreement across the full ECGI vest. Grey bars indicate electrodes excluded from analysis due to missing recordings or traces lacking identifiable QRS-T morphology.

Across the cohort, calibrated models achieved PCC≥0.6 at a median of 94.0% (IQR: 86.0 to 96.0%) and PCC≥0.8 at 82.0% (IQR: 70.0 to 88.0%) of the 50 representative electrodes (Fig 5A, 5B). Median PCC across all patients and sampled electrodes was 0.88 (IQR: 0.75 to 0.94). Individual patient performance ranged from 82% to 98% of electrodes achieving PCC≥0.6, with 12 patients exceeding 90% agreement, out of which 6 exceeded 95%.

To assess model performance beyond the calibration subset, best-fit parameter configuration for each patient was used to simulate BSPs at all 252 ECGI vest electrode locations. Calibration performance was preserved, with moderate-to-good agreement achieved across the cohort at a median of 94.0% (IQR: 91.6 to 96.8%) of electrodes and good agreement at 83.6% (IQR: 77.6 to 90.1%). Median *PCC* across the full vest was 0.89 (IQR: 0.80 to 0.94) (Fig 5C, 5D). The calibration performance of depolarisation versus repolarisation was also compared (Table 1). *PCC* were computed on the QRS segment and the T-wave in isolation, as well as on the full QRST waveform. Agreement on the QRS segment was systematically higher, indicating that depolarisation morphology was reproduced with higher fidelity than repolarisation. The T-wave only *PCC* was the lowest of the three, reflecting residual error in repolarisation morphology. It should be noted that for the electrodes with flat or near-isoelectric T-wave, *PCC* is dominated by baseline noise rather than waveform shape, yielding spuriously low values. The full QRST waveform *PCC* mitigates this by anchoring the correlation in the high-amplitude QRS complex, and is therefore retained as the primary repolarisation-inclusive metric.

**Table 1:** Calibration performance on the QRS segment, T-wave and the full QRST waveform, reported as median (IQR) across the cohort (*n* = 17).

Metric	QRS segment	T-wave	Full QRST waveform
***Sampled electrodes (n = 50)***			
Median *PCC*	0.91 (0.82 to 0.96)	0.79 (0.49 to 0.92)	0.88 (0.75 to 0.94)
% electrodes PCC≥0.6	96.0 (92.0 to 98.0)	82.0 (67.5 to 83.5)	94.0 (86.0 to 96.0)
% electrodes PCC≥0.8	84.0 (76.0 to 90.0)	63.0 (40.5 to 69.0)	82.0 (70.0 to 88.0)
***Full vest (n = 252)***			
Median *PCC*	0.92 (0.84 to 0.96)	0.83 (0.59 to 0.94)	0.89 (0.80 to 0.94)
% electrodes PCC≥0.6	96.0 (94.4 to 97.6)	83.5 (73.2 to 89.5)	94.0 (91.6 to 96.8)
% electrodes PCC≥0.8	86.9 (83.3 to 91.6)	69.3 (34.4 to 79.3)	83.6 (77.6 to 90.1)

At the per-patient level, consistency of calibration performance was quantified by the difference in match rate (Δ in %) between the full (252) and sampled (50) vest channels, which was positive for most patients (median Δ = 0.8%, IQR: ± 4.4%) (S9 Fig). Calibration quality on the sampled electrodes was thus closely matched by performance across the remaining unseen electrodes. This confirms that the 50-electrode subset sufficiently captured the relevant BSP information needed for model fitting and the calibrated parameters reproduced clinical signals consistently across the full torso surface.

**Fig 6:**
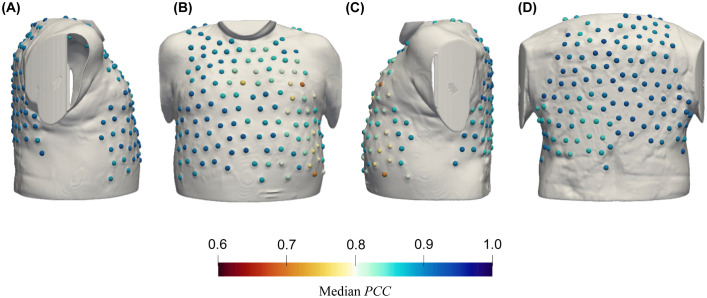
Spatial distribution of electrode-wise agreement between simulated and clinical body surface potentials. Cohort-level median Pearson correlation coefficients (PCCs) are projected onto a template ECGI vest geometry for four anatomical views: (A) right lateral, (B) anterior, (C) left lateral, and (D) posterior. Colour bar shows distribution of only *PCC* > 0.6 as no contiguous regions of poor agreement (*PCC* < 0.6) were observed.

Fig 6 shows the spatial distribution of electrode-wise agreement using cohort-level median *PCC*. A clear distinction was observed between far-field and near-field recording sites. Posterior and lateral torso surfaces, characterized by far-field potentials, showed consistently high correlation (PCC≥0.8). In contrast, localized regions of moderate agreement (0.8>PCC≥0.6) were confined to the left anterior thorax, corresponding to the precordial region directly overlying the ventricles. Contralateral (right) anterior surface maintained high correlation. Importantly, no contiguous regions of poor agreement (*PCC* < 0.6) were observed, supporting the overall stability and robustness of calibration.

**Fig 7:**
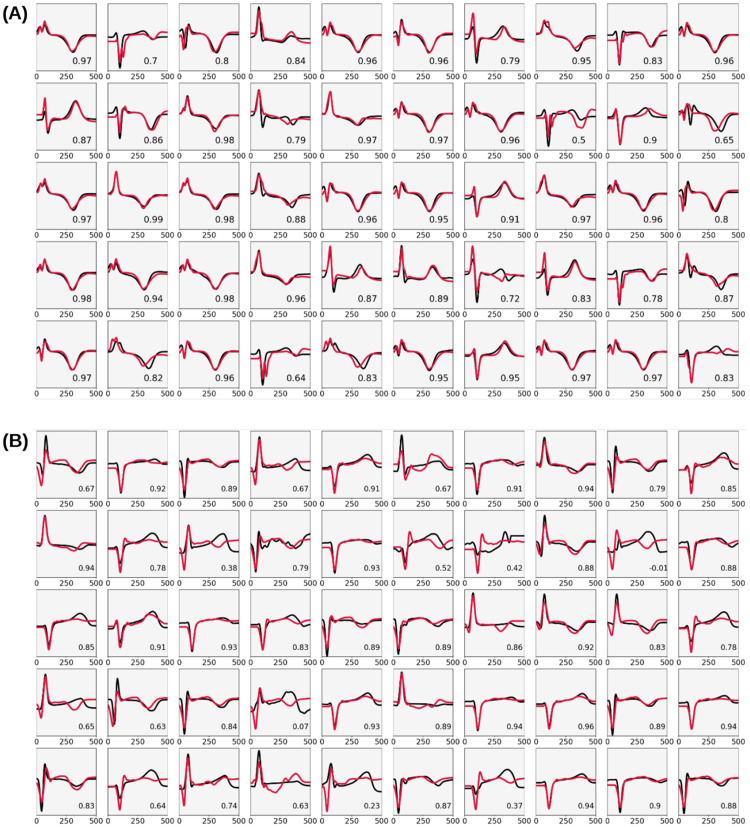
Representative higher- and lower-performing model calibration cases across the ECGI vest. Aligned clinical (black) and simulated (red) BSP waveforms are shown for their 50 sampled electrodes. Panel (A) shows a higher-performing patient and panel (B) a lower-performing patient, selected from the upper and lower ends of the cohort based on percentage agreement. All traces are displayed over an identical time window with consistent amplitude scaling.

To illustrate waveform-level model behaviour, representative higher- and lower-performing cases are reported in Fig 7. For the higher-performing case (Fig 7A), 98% of electrodes achieved PCC≥0.6, with good agreement observed for both depolarisation and repolarisation phases. QRS-complex and T-wave shape, polarity, and timing were well captured and consistently reproduced across diverse signal morphologies, indicating robust waveform-level calibration. In contrast, the lower-performing case (Fig 7B) exhibited marked spatial heterogeneity. While a significant portion of electrodes retained high fidelity (PCC≥0.8), a subset showed visible deviations in waveform morphology. Discrepancies were most pronounced in the T-wave, where simulated traces occasionally flattened repolarisation features or inverted signal polarity. The majority of the leads still preserved a physiologically interpretable signal structure. The spatial distribution of the 50 sampled electrodes used to calibrate these two patients is shown in S10 Fig.

To assess whether calibration quality depended on patient characteristics, associations between the percentage of sampled electrodes achieving PCC≥0.6 for the best-fit were examined against demographic and clinical variables (S11 and S12 Figs). No statistically significant associations were observed with age, sex, BSA, maximum LV wall thickness, LA diameter, arrhythmic history, or ESC SCD risk score (all *p* > 0.05). A moderate negative Pearson correlation (−0.58, *p* = 0.014) was observed between LVOTO gradient and sampled-electrode match percentage. However, this association was not confirmed using rank-based Spearman correlation (ρ=−0.49, *p* = 0.069) and did not survive multiple testing corrections. Overall, no major phenotypic markers were significantly associated with calibration performance in this cohort. Given the modest sample size (*n* = 17), the absence of statistically significant associations should be interpreted cautiously.

### Model validation under pacing

Model generalization to unseen activation patterns was evaluated using RV apical pacing, following the trend-based pacing validation protocol detailed in the Methods.

**External Cohort Pacing Responses.** Global depolarisation was markedly prolonged, with QRS duration ($QRS_{d}$) increasing by a median of 54 ms (IQR: 33–69 ms, *p* < 0.001). At the local lead level (V1-V8), ventricular activation time ($V_{d}$) increased by a median of 35.3 ms (IQR: 25.7 to 42.4 ms, *p* < 0.001). Consistent with delayed bulk activation, the temporal centroid of the QRS complex ($QRS_{max,center}$) shifted later by a median of 44.2 ms (IQR: 29.6 to 56.2 ms, *p* < 0.001).

Repolarisation exhibited a systematic reduction in activation-recovery interval (*ARI*), with a median decrease of 18.8 ms (IQR: -49.0 to -10.4 ms, *p* = 0.001). T-wave onset ($T_{on}$) and offset ($T_{off}$) exhibited significant (*p* < 0.05) delays by 49.7 ms (IQR: 4.5 to 57.3 ms) and 44.6 ms (IQR: 30.5 to 61.3 ms) respectively, reflecting pacing-induced shifts in overall repolarisation timing. In contrast, repolarisation time (*RT*) and time-to-peak T-wave ($T_{max}$) showed no statistically significant (*p* = 0.64 and *p* = 0.07, respectively) or consistent directional changes and were therefore excluded from subsequent analysis.

**Simulated Pacing Responses.** Using the selected metrics, the RV apical pacing protocol was applied to the calibrated EP models. Changes in retained ECG metrics were quantified relative to the corresponding sinus simulations. As BSP recordings do not provide standard V1-V8 lead ECGs, lead-specific metrics were derived from anatomically corresponding vest electrodes.

**Fig 8:**
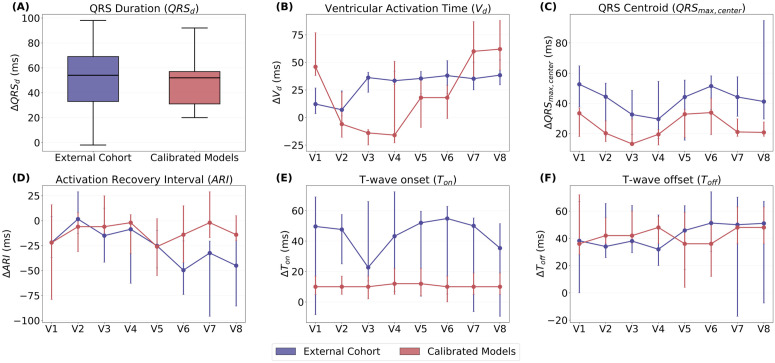
Pacing-induced changes in ECG-derived depolarisation and repolarisation metrics for an external clinical cohort and calibrated digital twins. Comparison of pacing-induced changes (Δ = pacing - sinus) between an independent external HCM validation cohort (blue, *n* = 13) and calibrated digital twins (red, *n* = 17). **(A)** Global QRS duration changes ($\Delta QRS_{d}$) shown as box-and-whisker plots. **(B-F)** Lead-specific changes across precordial leads V1-V8 displayed as median with IQR error bars: (B) ventricular activation time ($\Delta V_{d}$), **(C)** QRS temporal centroid ($\Delta QRS_{max,center}$), (D) activation-recovery interval (ΔARI), **(E)** T-wave onset ($\Delta T_{on}$), and **(F)** T-wave offset ($\Delta T_{off}$).

Pacing responses in the calibrated models closely reproduced the directional trends observed in the external clinical cohort (Fig 8). Global depolarisation prolongation was robustly reproduced, with $QRS_{d}$ increasing by a median of 52 ms (IQR: 31–61 ms). Local activation was similarly delayed, with $V_{d}$ increasing by a median of 25.0 ms (IQR: 8.5 to 41.5 ms), with increased inter-lead variability, and $QRS_{max,center}$ shifting rightward by a median of 20.9 ms (IQR: 17.2 to 30.5 ms), indicating delayed bulk ventricular activation.

Repolarisation responses exhibited greater inter-lead variability, consistent with their known sensitivity to activation sequence and waveform morphology. Nonetheless, the calibrated models consistently reproduced key qualitative pacing signatures observed clinically. *ARI* preserved the directionality and spatial pattern of change with a median reduction of 10.0 ms (IQR: -21.5 to -1.0 ms) across precordial leads. T-wave timings were also delayed under pacing, with $T_{on}$ increasing by a median of 12 ms (IQR: 8–17 ms) and $T_{off}$ by a median of 42 ms (IQR: 36–55 ms). Consistent with the external cohort, *RT* and $T_{max}$ showed no statistically significant pacing-induced change in the calibrated models (*p* = 0.78 and *p* = 0.07, respectively).

Overall, EP models calibrated exclusively to intrinsic rhythm generalised well to RV apical pacing by reproducing the dominant physiological signatures of altered ventricular activation and repolarisation. Strong agreement was observed for depolarisation-based metrics, while repolarisation metrics exhibited consistent qualitative trends with increased variability.

### Analysis of calibrated parameters

To ensure calibration targeted parameters with observable influence on BSP morphology, the full 122-parameter model was first screened using SA (see Methods). This reduced the problem to phase-specific subsets of influential parameters. Depolarisation calibration involved 18 parameters per patient (IQR: 17–19), while repolarisation required 23 parameters (IQR: 19–26). The staged strategy reduced the effective calibration space to 41 parameters per patient (IQR: 37–44). Sensitivity rankings and retained parameter sets for each stage are reported in S4-S6 Figs.

HM-based calibration substantially reduced the joint EP parameter space for both depolarisation and repolarisation stages. The final calibration wave retained a median of 6.64% (IQR: 3.58 to 19.43%) of the initial depolarisation search space as plausible. Repolarisation calibration constrained the viable parameter space to 12.48% (IQR: 8.33 to 25.65%) of the prior space. Overall, HM ruled out 87–93% of initial parameter combinations (median 93% for QRS, 88% for T-wave), confirming that clinical BSPs impose strong constraints on admissible joint parameter configurations, though the degree of constraint varied substantially across patients.

To assess whether calibration converged to physiologically meaningful solutions, best-fit distributions of key EP parameters were examined across patients for the QRS-complex (S13 Fig) and the T-wave (S14 Fig). Despite broad uniform priors (see Supplementary Tables), calibrated parameter values converged to physiologically plausible values. Baseline LV CV ($CV_{f,LV}$) was 0.62 m/s (IQR: 0.52 to 0.74 m/s), with apico-basal and transmural gradients of 1.31 (IQR: 1.11 to 1.47) and 0.87 (IQR: 0.76 to 0.94), respectively. The SE layer exhibited faster conduction, with a scaling factor ($CV_{f}^{SE}/CV_{f}$) of 4.58 (IQR: 2.94 to 6.29) times bulk myocardium velocity. Anisotropy ratios constrained at 0.47 (IQR: 0.43 to 0.49), corresponding to transverse conduction velocity approximately half of longitudinal velocity. Early activated fascicle locations and timings showed moderate variability across patients, reflecting the need for patient-specific tuning of the HPS.

Accurate T-wave reproduction required patient-specific tuning of repolarisation gradients across multiple ionic currents. The apico-basal gradient of the rapid delayed rectifier potassium channel conductance ($G_{Kr,LV}$) showed a median scaling of 1.09 (IQR: 0.95 to 1.59), while transmural gradients were at 1.11 (IQR: 0.90 to 1.48). The background potassium channel conductance ($G_{Kb,LV}$) displayed similar apico-basal gradients of 1.38 (IQR: 0.71 to 2.99) but with notably broader interquartile ranges, indicating greater variability in its role across patients. The late sodium channel conductance ($G_{NaL,LV}$) showed pronounced apico-basal gradients (median: 1.85, IQR: 1.32 to 2.07) and moderate transmural heterogeneity (1.29, IQR: 1.04 to 1.49). The L-type calcium current permeability ($P_{Ca,LV}$) exhibited the strongest gradients among all ionic parameters, with apico-basal scaling of 2.35 (IQR: 1.73 to 2.44) and transmural scaling of 2.19 (IQR: 1.80 to 2.68), reflecting its critical role in action potential plateau and duration. The sodium-calcium exchanger ($G_{NCX,LV}$) showed moderate spatial gradients (apico-basal: 1.53, IQR: 1.04 to 1.84; transmural: 1.42, IQR: 1.26 to 1.87), contributing to regional differences in calcium handling and repolarisation dynamics.

**Fig 9:**
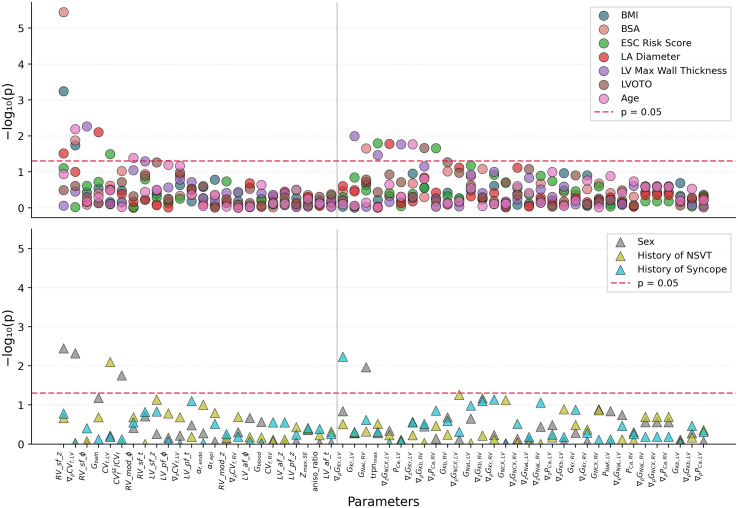
Correlation between calibrated electrophysiological parameters and clinical demographics. Manhattan plots summarising associations between patient-specific calibrated EP parameters and continuous (top) and categorical (bottom) clinical variables. Parameters are grouped by QRS-related vs repolarisation-related, separated by the vertical divider. The red dashed line indicates nominal significance (*p* = 0.05).

To explore whether calibrated parameters reflect clinical markers, associations were examined between best-fit model parameters and available demographic and clinical variables. Parameter-phenotype associations were assessed using Spearman correlation (ρ) for continuous variables, and Mann-Whitney U tests for categorical comparisons. Comprehensive screening identified 30 nominal associations (*p* < 0.05), comprising 24 continuous (S10 Table) and 6 categorical comparisons (S11 Table), as summarised in Fig 9.

Among continuous variables, body size metrics (BSA and BMI) showed correlations with parameters governing spatial fascicle positioning and CV gradients. Negative correlation was observed between BSA and RV septal fascicle apico-basal coordinate (ρ=−0.91, *p* < 0.001) and both BSA and BMI with the transmural LV velocity gradient (ρ=−0.62, *p* = 0.013 and ρ=−0.60, *p* = 0.018, respectively). Age exhibited associations with multiple conduction and repolarisation-related parameters, including a negative correlation with transmural LV velocity gradient (ρ=−0.67, *p* < 0.01) and a positive correlation with LV apico-basal gradient of $G_{Kr}$ (ρ=0.6 and *p* = 0.017). Structural cardiac markers, including maximum LV wall thickness, and LA diameter, were correlated with ionic conductances. LV wall thickness showed negative associations with baseline values of $G_{Kr}$ (ρ=−0.64, *p* = 0.010) and $P_{Ca}$ (ρ=−0.60, *p* = 0.017) in the LV, and positive with the RV septal fascicle rotational coordinate (ρ=0.68 and *p* = 0.005). LA diameter correlated with conductance of the torso cavity (ρ=0.66, *p* = 0.008), and apico-basal gradient of $G_{NCX}$ (ρ=0.61, *p* = 0.017). ESC SCD risk score showed modest correlations with $CV_{f,LV}$ (ρ=0.55, *p* = 0.03).

Categorical differences were observed for a subset of parameters. Patients with a history of NSVT showed higher calibrated $CV_{f,LV}$ (*U* = 4.000, *p* = 0.008), while patients with a history of syncope exhibited lower transmural gradients of $G_{Kr,LV}$ (*U* = 42.000, *p* = 0.006). Additional differences were observed between men and women for parameters including $CV_{f}^{SE}/CV_{f}$, $\nabla_{\rho }CV_{f,LV}$, RV_sf_z and $G_{NaL,RV}$, with *p*-values ranging from 0.004 to 0.018. Detailed parameter associations are visualised in S15 and S16 Figs.

## Discussion

This study presents a comprehensive framework for constructing EP digital twins of HCM by integrating multimodal clinical imaging with emulator-based Bayesian HM. Across a heterogeneous cohort, the approach achieved high-fidelity reproduction of BSPs recorded using an ECGI vest, with calibrated models converging to physiologically plausible parameter values. Importantly, parameter sets inferred from sinus rhythm generalized robustly to RV pacing without additional tuning, reproducing clinically observed trends in depolarisation and repolarisation. Together, these results indicate that the proposed framework captures intrinsic patient-specific electrophysiological properties rather than overfitting surface-level signal morphology.

### Calibration framework

Unlike previous model personalization studies relying on standard 12-lead ECG [27,41], our framework leverages 252-electrode BSP data to directly constrain the high-dimensional EP parameter space. The advantages of 252-lead BSPs over 12-lead ECG for model personalization are supported by multiple validation studies. Kania et al. (2019) [42] reported improved detection of ischemic heart disease during exercise testing using BSP mapping. In silico pace mapping studies using 252-electrode ECGI vests further reported an improvement in pacing-site localization accuracy relative to 12-lead ECG [43]. Moreover, recent work by Joy et al. (2024) [11] showed that CMR-guided ECGI revealed spatially heterogeneous EP abnormalities that were absent in 12-lead ECGs, highlighting limitations of conventional surface ECG in HCM.

While BSPs provide substantially richer spatial information than 12-lead ECG, this density introduces challenges in parameter identifiability and computational cost given ≈126,000 data points per beat. To address this, targeted dimensionality reduction was applied at signal and parameter levels (see Methods). LOOCV used to select the cluster count (*k* = 50) also quantified the BSP data available for calibration as a function of electrode density. At the patient level, individual elbow locations clustered between *k* = 20–50 with a median of *k* = 30 (IQR: 30–40), reflecting modest inter-patient variability in *k* required to reconstruct held-out signals. At the cohort level, the elbow of the median curve occurred at *k* = 50, with a reconstruction error within 21% of the asymptotic value at *k* = 100 (cohort medians of 2.96 and 2.44 respectively). Notably, cross-validated reconstruction error at *k* = 10 was approximately 50% higher than at *k* = 50 (cohort medians of 4.46 and 2.96 respectively). Reducing the calibration target to the 12-lead regime would therefore degrade its representativeness of the full 252-electrode vest and limit the consistency of calibration across unsampled electrodes.

With the reduced parameter space, calibration employed Bayesian HM rather than traditional optimization techniques. HM iteratively rules out implausible parameter regions while retaining physiologically plausible ensembles, an approach well-suited to cardiac EP models with inherent parameter non-identifiability and model-data discrepancy. Previous studies have demonstrated the effectiveness of HM for calibrating cardiac and biophysical models under similar constraints [44–46].

A central consideration for biophysically detailed EP models is parameter identifiability. Multiple parameter combinations can produce similar BSP morphologies, particularly within physiologically correlated subsets. HM is well-suited to this regime because it does not seek a unique point estimate but instead returns the NROY ensemble. The framework therefore reports a constrained, physiologically plausible parameter region rather than a single best-fit point. Individual parameter values within NROY should be interpreted as samples from this region rather than as uniquely identified physiological quantities.

Sequential calibration (QRS then T-wave) further improved tractability, reflecting distinct physiological timescales supported by SA identifying largely separate parameter subsets (S5 and S6 Figs). While this strategy substantially reduced computational cost, it may underestimate interactions between depolarisation and repolarisation mechanisms. Additionally, T-wave calibration required more iterations than QRS and accounted for the majority of residual waveform discrepancies in lower-performing cases, consistent with the distinct physiological complexity of repolarisation. Quantitatively, agreement on the QRS segment was systematically higher than on the T-wave (Table 1). Simultaneous calibration would be theoretically preferable but remains computationally prohibitive at the organ scale.

### Model fidelity and spatial patterns of agreement

Calibrated models achieved PCC≥0.6 at median of 94.0% (IQR: 86.0 to 96.0%) of representative electrodes, with median PCC of 0.88 (IQR: 0.75 to 0.94), comparing favourably with published benchmarks [47,48]. Importantly, when extending to the full 252-electrode vest without retuning, performance improved slightly by a median of +0.8%, indicating that calibration captured intrinsic tissue properties rather than channel-specific signal features of the selected electrode subset.

The spatial distribution pattern of electrode-wise agreement was physiologically interpretable and in agreement with the biophysics of volume conduction (Fig 6). Far-field electrodes on posterior and lateral torso surfaces consistently exhibited higher correlation (PCC≥0.8). As these BSPs integrate electrical activity over large anatomical volumes, intervening tissue layers (myocardium, blood, lungs, muscle, fat, skin) act as low-pass filters, attenuating sharp gradients and smoothing waveform morphology [49]. This makes far-field signals less sensitive to localized EP variations and easier to reproduce.

In contrast, regions of comparatively lower agreement (0.8>PCC≥0.6) were localized to the left anterior precordial region directly overlying the ventricles, where near-field electrodes capture sharper spatial gradients with intrinsic sensitivity to localized EP and anatomical heterogeneities. This is compounded by the steep spatial potential gradient across the precordial transition zone, where QRS polarity changes over a short distance. Consequently, small spatial mismatches in registration, electrode positioning, and fiber orientation translate into disproportionately large waveform discrepancies. Prior studies confirm that these precordial leads, particularly V3 and V4, exhibit higher reconstruction errors and stronger conductivity anisotropy sensitivity [50,51]. They are also more sensitive to changes in heart positioning between BSP and imaging measurements, making accurate reproduction more challenging.

Notably, electrodes on the right anterior torso maintained high correlation despite their anterior position, and no contiguous regions of poor agreement (*PCC* < 0.6) were observed. Overall, the observed spatial patterns of agreement indicate that model performance is governed by physiologically plausible sensitivities rather than global inaccuracies.

### Pacing validation

To evaluate whether models calibrated exclusively to sinus rhythm generalized to altered activation patterns, RV apical pacing was simulated without additional parameter tuning (see Methods). Validation assessed whether simulated pacing-induced changes in established ECG-derived EP markers reproduced characteristic physiological trends observed in the independent external HCM cohort. This approach aligns with established validation theory distinguishing direct, data-matched validation from context-of-use-driven or phenomenon-based validation [52]. RV apical pacing produces well-characterized effects such as, QRS prolongation, delayed LV activation, and systematic shifts in repolarisation timing, which provide a suitable benchmark. Reproducing these signatures with comparable magnitudes constitutes evidence of model generalization beyond the calibration domain.

$QRS_{d}$ was prolonged by 54 ms in the external cohort compared to 52 ms in the calibrated digital twins (Fig 8A). $V_{d}$ increased by 35.3 ms in the external cohort, with digital twins showing an increase of 25.0 ms (Fig 8B). While this median increase was slightly smaller, the preserved directionality and broad IQR suggest that the models captured spatially heterogeneous pacing responses, with variability driven by patient-specific anatomy and lead sampling. *QRS*_max,center_ was delayed by 44.2 ms in the external cohort and 20.9 ms in the digital twins (Fig 8C). The reduced magnitude likely reflects a combination of methodological differences in centroid computation and the use of conduction properties calibrated during sinus rhythm. Despite this underestimation, the consistent positive shift across all patients supports qualitatively correct generalization of bulk ventricular activation delay.

Repolarisation responses to pacing were more variable, consistent with their greater physiological complexity. ARI decreased by a median of 18.8 ms in the external cohort. Digital twins preserved the directional trend, with a median ARI reduction of 10.0 ms (Fig 8D). ARI shortening under pacing reflects interactions between early- and late-activated regions and altered mechanical loading during dyssynchronous contraction. Preservation of the trend despite reduced magnitude indicates that the calibrated ionic models qualitatively captured these coupled effects. *T*_on_ and *T*_off_ were significantly delayed in the external cohort. Digital twins reproduced both delays (Fig 8E-8F), with *T*_off_ showing particularly strong agreement (42 ms simulated vs. 44.6 ms observed), while *T*_on_ exhibited a smaller but consistent delay. RT and *T*_max_ showed no consistent or statistically significant changes in the external cohort and were therefore excluded from validation.

The close agreement in median values and overlapping interquartile ranges indicates that the digital twins captured not only the expected direction and magnitude but also the inter-patient variability in pacing response. This suggests that constrained parameters captured intrinsic tissue electrophysiological properties rather than merely reproducing surface BSP morphology.

### Parameter-phenotype associations

Among the explored correlations, 30 were found to be statistically significant. The direction and mechanistic nature of several exploratory trends aligned with independent clinical, experimental, and computational evidence. Age negatively correlated with transmural LV velocity gradient. This aligns with progressive lateral gap junction uncoupling [53], age-dependent collagen accumulation [54], and energetic substrate alterations [55] that reduce transmural heterogeneity. Further, positive correlation of age with the apico-basal gradient of $G_{Kr}$, is explained mechanistically by recent ECGI findings that apico-basal RT dispersion inverts with age [56], as reduced apical $I_{Kr}$ (or enhanced basal $I_{Kr}$) directly prolongs local RT [57,58]. Convergence between clinical RT measurements and our calibrated conductance gradients validates age-driven repolarisation remodelling via systematic potassium redistribution.

BSA and BMI also showed negative correlations with transmural LV velocity gradient, consistent with obesity-associated conduction slowing [59,60] via adipose infiltration promoting non-uniform propagation [61]. BSA demonstrated a negative correlation with the apico-basal coordinate of the RV septal fascicle breakthrough site, indicating a more apical onset of RV activation in larger subjects. This finding captures the geometric consequences of cardiac allometry, where ventricular dimensions scale non-linearly with body size and suggests that our simplified fascicular model successfully preserves the geometric fidelity of the HPS relative to the dilated ventricular geometries associated with larger body size.

Disease severity markers (larger LA diameter, greater LV wall thickness, higher ESC SCD risk scores) were associated with more pronounced ionic remodelling patterns. Sex-specific differences were observed, with $CV_{f}^{SE}/CV_{f}$ and transmural gradients of CV concentrated in the lower and upper bounds respectively for women. On the other hand, men displayed greater inter-subject variability with calibrated parameter values spread across prescribed ranges. Similar patterns were observed when comparing patients grouped by history of NSVT and syncope. Individuals with a history of NSVT calibrated to upper quartiles of $CV_{f,LV}$, whereas those without showed greater variability. History of syncope drove transmural gradient of $G_{Kr}$ to be < 1, meaning the value of $G_{Kr}$ at the apex is higher than that on the base. Individuals without a history of syncope calibrated to gradients > 1, implying base > apex.

Given the relatively small cohort size, the absence of additional statistically significant associations should be interpreted cautiously. Likewise, the asymmetry between QRS and T-wave calibration suggest that conduction-related parameters are inferred with greater confidence than repolarisation-related parameters. Altogether, these findings indicate that while patient-specific BSP morphology provides primary constraints on electrophysiological parameter calibration, clinical covariates may exert secondary influences on specific parameters, particularly those governing conduction system geometry and ionic heterogeneity.

### Limitations

To improve model fitting and calibration performance, we identify limitations that should be addressed in future work. Small misalignments in co-registration of CMR-derived cardiac anatomy and CT-based torso geometry can introduce position-dependent errors. Although registration accuracy was within acceptable bounds [62,63], small translational errors can affect reproduction of near-field electrode signals.

The simplified fascicular-based HPS representation cannot fully capture patient-specific conduction system complexity. While this approach effectively modulates the rapid endocardial spread of activation and has been validated for 12-lead ECG synthesis [27], it lacks the ability to represent disease-induced alterations in Purkinje density and arborization [64]. Similarly, EP heterogeneity was prescribed as linear gradients along the apico-basal and transmural directions. This parametrisation captures dominant physiological trends but cannot represent finer regional or non-monotonic spatial patterns of conduction and repolarisation that may be present in HCM.

The absence of patient-specific diffusion tensor imaging (DTI), constrained myocardial fiber orientation to deviations in fiber and sheet angles around a rule-based template. Comparative studies of Laplace-Dirichlet rule-based methods report fiber-angle differences of 5° to 10° between implementations, with the largest discrepancies occurring in mid-transmural layers [24,65]. Moreover, DTI-derived epicardial fiber orientations exhibit increased directional variability that cannot be captured by smooth rule-based formulations.

Myocardial fibrosis and scar were not explicitly incorporated into the anatomical models. In HCM, fibrosis is present in approximately 60–80% of patients, with extent ranging from <5% to >20% of left ventricular mass [66,67]. Its distribution is spatially heterogeneous, with predilection for the interventricular septum and regions of maximal hypertrophy, areas that project strongly onto precordial electrodes. Fibrotic tissue alters both depolarisation (slowed conduction, increased anisotropy, zig-zag pathways) and repolarisation (altered action potential morphology and increased dispersion). Although the present framework implicitly models some of these effects through calibrated regional conduction velocities and ionic gradients, such phenomenological adjustments cannot reproduce the microstructural complexity of fibrotic remodelling. Explicit integration of LGE-CMR or T1-mapping data would likely improve near-field signal reproduction, particularly in patients with extensive scar burden.

It is important to acknowledge that a trend-based validation approach is inherently less stringent than direct patient-matched validation. Use of an external cohort assumes broadly similar pacing responses across patients with HCM, an assumption that may not hold for extreme phenotypes. In addition, the modest external cohort size (*n* = 13) limits precision, with the reported interquartile ranges reflecting substantial biological variability. Differences between simulated and clinical pacing sites may also contribute, as pacing location was standardized in simulations but may have varied slightly in clinical practice, where small deviations in lead placement can alter activation patterns. Finally, validation was restricted to a single pacing configuration (RV apical pacing). More comprehensive assessment of generalization would require evaluation under alternative pacing sites and additional physiological perturbations.

## Conclusion

This study established a comprehensive framework for developing patient-specific EP models of HCM using multimodal clinical imaging and emulator-based Bayesian calibration. Applied to 17 HCM patients, the workflow achieved high-fidelity reproduction of BSPs, with models converging to physiologically realistic parameter values. Calibrated models generalized robustly to RV pacing without additional tuning, reproducing clinical pacing responses including QRS prolongation, activation delays, and repolarisation changes. Exploratory parameter-phenotype associations showed biologically plausible trends including age-related conduction and repolarisation changes, body size effects on tissue properties, and wall thickness associations with ionic remodelling. These preliminary findings merit investigation in larger cohorts powered to detect moderate-effect associations.

The validated framework provides a foundation for mechanistic investigation of HCM electrophysiology, risk stratification enhancement, and in silico testing of therapeutic interventions. Future directions include integration of fibrosis imaging, patient-specific fiber architecture, larger multi-center cohorts for definitive parameter-phenotype studies, and clinical validation against longitudinal outcomes. Ultimately, patient-specific models hold promise for precision medicine in HCM, enabling individualized risk assessment and treatment optimization grounded in mechanistic understanding of each patient’s electrophysiological substrate.

## Supporting information

S1 Table Anatomical regions and their corresponding tags in the whole-torso finite element mesh. (PDF)

S2 Table Myocardial fibre and sheet angle parameters. (PDF)

S3 Table (A) Fixed parameters for early activation sites (root points) of the fascicular HPS. (B) Variable parameters for early activation sites (root points) of the fascicular HPS. (PDF)

S4 Table Myocardial conduction velocities. (PDF)

S5 Table HCM-specific ionic remodelling parameters. (PDF)

S6 Table Ion channel and transporter genes exhibiting regional expression gradients in human ventricles. (PDF)

S7 Table Single-cell ToR-ORd-dynCl model parameters. (PDF)

S8 Table Conductivities for all non-myocardial regions in the torso. (PDF)

S9 Table ECG-derived markers used for pacing analysis. (PDF)

S10 Table Associations between continuous clinical variables and calibrated parameters. (PDF)

S11 Table Associations between categorical clinical variables and calibrated parameters. (PDF)

S1 Fig Representative examples of CMR-to-CT heart registration. (PDF)

S2 Fig Mapping of ECGI vest electrodes to the torso mesh and selection of the reference electrode. (PDF)

S3 Fig Fascicular-based modelling of the His-Purkinje system (HPS). (PDF)

S4 Fig Global sensitivity analysis of the ToR-ORd-dynCl ionic model. (PDF)

S5 Fig QRS-complex parameter screening across patients. (PDF)

S6 Fig T-wave parameter screening across patients. (PDF)

S7 Fig Emulator-based Bayesian history matching framework. (PDF)

S8 Fig Calibration computational cost quantified as history matching (HM) iterations or “waves”. (PDF)

S9 Fig Consistency of calibration performance between representative and full vest electrode sets. (PDF)

S10 Fig Spatial distribution of sampled electrodes for representative higher- and lower-performing cases. (PDF)

S11 Fig Association between calibration match percentage and continuous demographic and clinical variables. (PDF)

S12 Fig Calibration match percentage stratified by categorical demographic and clinical variables. (PDF)

S13 Fig Physiological distributions of calibrated ventricular conduction parameters. (PDF)

S14 Fig Distributions of calibrated repolarisation-related ionic parameters. (PDF)

S15 Fig Exploratory associations between calibrated parameters and categorical clinical phenotypes. (PDF)

S16 Fig Exploratory continuous parameter-phenotype associations. (PDF)

S1 Appendix Supplementary methods. Detailed descriptions of: (1) medical image segmentation and multimodal registration; (2) the reaction-eikonal framework; (3) the fascicular His-Purkinje system; (4) the framework to assign EP model heterogeneity; (5) scaling factor and estimation of variability ranges; (6) myocardial conduction velocity and conductivities; (7) signal processing; (8) linear-regression-based sensitivity analysis; (9) emulator training; and (10) Bayesian history matching.
(PDF)
