# Supplementary material for: Body surface potential driven personalisation of electrophysiological digital twins in hypertrophic cardiomyopathy
Source: PLoS Comput Biol. 2026 Jul 27;22(7):e1014555. doi: 10.1371/journal.pcbi.1014555 (PMC13432148; doi:10.1371/journal.pcbi.1014555)
Supplement: S1 Table — (PDF) [file pcbi.1014555.s001.pdf]

S1 Table. Anatomical regions and their corresponding tags in the whole-torso finite element mesh. Each patient-specific mesh comprised 23 distinct anatomical regions. Region tags were used to assign tissue-specific conductivities and electrophysiological properties for simulations.

Region Tag	Anatomical Structure
1	Torso cavity
2	Skin
3	Bones
4	Spinal cord
5	Kidneys
6	Pancreas
7	Spleen
8	Liver
9	Stomach
10	Lungs
11	Inferior vena cava
12	Superior vena cava
13	Pulmonary artery
14	Pulmonary artery wall
15	Aorta
16	Right atrium
17	Right atrial wall
18	Left atrium
19	Left atrial wall
20	Right ventricle
21	Left ventricle
22	Right ventricular wall
23	Left ventricular myocardium
