# Supplementary material for: Body surface potential driven personalisation of electrophysiological digital twins in hypertrophic cardiomyopathy
Source: PLoS Comput Biol. 2026 Jul 27;22(7):e1014555. doi: 10.1371/journal.pcbi.1014555 (PMC13432148; doi:10.1371/journal.pcbi.1014555)
Supplement: S2 Table — (PDF) [file pcbi.1014555.s002.pdf]

S2 Table. Myocardial fibre and sheet angle parameters. Parameter definitions, baseline values and ranges of variation for endocardial and epicardial fibre and sheet angles used for rule-based fibre orientation assignment via the Laplace-Dirichlet method [1]. All angles are reported in degrees and were adopted from previous clinical and experimental studies [2] [3] [4] [5] [6].

**Parameter**	**Description**	**Baseline**	**Range**
$\alpha_{f,\text{endo}}$	Endocardial fibre angle	+60	$[+30, +120]$
$\alpha_{f,\text{epi}}$	Epicardial fibre angle	−60	$[-120, -30]$
$\beta_{s,endo}$	Endocardial sheet angle	−65	$[-95, -35]$
$\beta_{s,epi}$	Epicardial sheet angle	+25	$[-10, +60]$

References

1. Bayer JD, Blake RC, Plank G, Trayanova NA. A novel rule-based algorithm for assigning myocardial fiber orientation to computational heart models. Annals of biomedical engineering. 2012;40(10):2243-54. doi:https://doi.org/10.1007/s10439-012-0593-5.

2. Gillette K, Gsell MAF, Prassl AJ, Karabelas E, Reiter U, Reiter G, et al. A Framework for the generation of digital twins of cardiac electrophysiology from clinical 12-leads ECGs. Medical Image Analysis. 2021;71:102080. doi:https://doi.org/10.1016/j.media.2021.102080.

3. Greenbaum R, Ho SY, Gibson D, Becker A, Anderson R. Left ventricular fibre architecture in man. Heart. 1981;45(3):248-63. doi:https://doi.org/10.1136/hrt.45.3.248.

4. Arts T, Costa KD, Covell JW, McCulloch AD. Relating myocardial laminar architecture to shear strain and muscle fiber orientation. American Journal of Physiology-Heart and Circulatory Physiology. 2001;280(5):H2222-9. PMID: 11299225. doi:10.1152/ajpheart.2001.280.5.H2222.

5. Wong J, Kuhl E. Generating fibre orientation maps in human heart models using Poisson interpolation. Computer methods in biomechanics and biomedical engineering. 2014;17(11):1217-26. doi:https://doi.org/10.1080/10255842.2012.739167.

6. Li P, Sun A, Guo C, Peng Z, Wang C. Effects of orientation of myocardial fibers on the contractility of left ventricle. Journal of the Mechanical Behavior of Biomedical Materials. 2025;168:107025. doi:https://doi.org/10.1016/j.jmbbm.2025.107025.
