# Supplementary material for: Body surface potential driven personalisation of electrophysiological digital twins in hypertrophic cardiomyopathy
Source: PLoS Comput Biol. 2026 Jul 27;22(7):e1014555. doi: 10.1371/journal.pcbi.1014555 (PMC13432148; doi:10.1371/journal.pcbi.1014555)
Supplement: S3 Table — (PDF) [file pcbi.1014555.s003.pdf]

S3A Table. Fixed parameters for early activation sites (root points) of the fascicular HPS. Baseline values of transmural [ρ from endocardium (0) to epicardium (1)] and chamber [ν LV (-1) vs RV (1)] coordinates.

Parameter	Description	Baseline
LV_sf_ρ	Transmural coordinate for LV septal fascicle	0
LV_sf_ν	Chamber coordinate for LV septal fascicle	-1
LV_pf_ρ	Transmural coordinate for LV posterior fascicle	0
LV_pf_ν	Chamber coordinate for LV posterior fascicle	-1
LV_af_ρ	Transmural coordinate for LV anterior fascicle	0
LV_af_ν	Chamber coordinate for LV anterior fascicle	-1
RV_sf_ρ	Transmural coordinate for RV septal fascicle	1
RV_sf_ν	Chamber coordinate for RV septal fascicle	-1
RV_mod_ρ	Transmural coordinate for RV moderator band	0
RV_mod_ν	Chamber coordinate for RV moderator band	1

S3B Table. Variable parameters for early activation sites (root points) of the fascicular HPS. Ranges of variation were based on previous studies [1] [2] [3].

Parameter	Description	Baseline	Range
LV_sf_z	Apico-basal coordinate for LV septal fascicle	0.66	$[0.2, 0.85]$
LV_sf_ϕ	Rotational coordinate for LV septal fascicle	-0.14	$[-1.57, 1.57]$
LV_sf_t	Activation timing for LV septal fascicle (in ms)	1.0	$[0, 15]$
LV_pf_z	Apico-basal coordinate for LV posterior fascicle	0.70	$[0.2, 0.85]$
LV_pf_ϕ	Rotational coordinate for LV posterior fascicle	-2.03	$[-3.14, 0]$
LV_pf_t	Activation timing for LV posterior fascicle (in ms)	0.4	$[0, 15]$
LV_af_z	Apico-basal coordinate for LV anterior fascicle	0.65	$[0.2, 0.85]$
LV_af_ϕ	Rotational coordinate for LV anterior fascicle	2.01	$[0, 3.14]$
LV_af_t	Activation timing for LV anterior fascicle (in ms)	2.5	$[0, 15]$
RV_sf_z	Apico-basal coordinate for RV septal fascicle	0.80	$[0.2, 0.85]$
RV_sf_ϕ	Rotational coordinate for RV septal fascicle	-0.5	$[-1.2, 1.2]$
RV_sf_t	Activation timing for RV septal fascicle (in ms)	5.0	$[0, 30]$
RV_mod_z	Apico-basal coordinate for RV moderator band	0.25	$[0.2, 0.85]$
RV_mod_ϕ	Rotational coordinate for RV moderator band	0.0	$[-1.57, 1.57]$
RV_mod_t	Activation timing for RV moderator band (in ms)	10.0	$[0, 30]$

References

1. DURRER D, DAM RTV, FREUD GE, JANSE MJ, MEIJLER FL, ARZBAECHER RC. Total Excitation of the Isolated Human Heart. Circulation. 1970;41(6):899-912. doi:10.1161/01.CIR.41.6.899.

2. Massing GK, James TN. Anatomical configuration of the His bundle and bundle branches in the human heart. Circulation. 1976;53(4):609-21. doi:10.1161/01.CIR.53.4.609.

3. Atkinson A, Inada S, Li J, Tellez JO, Yanni J, Sleiman R, et al. Anatomical and molecular mapping of the left and right ventricular His–Purkinje conduction networks. Journal of Molecular and Cellular Cardiology. 2011;51(5):689-701. doi:https://doi.org/10.1016/j.yjmcc.2011.05.020.
