# Supplementary material for: Body surface potential driven personalisation of electrophysiological digital twins in hypertrophic cardiomyopathy
Source: PLoS Comput Biol. 2026 Jul 27;22(7):e1014555. doi: 10.1371/journal.pcbi.1014555 (PMC13432148; doi:10.1371/journal.pcbi.1014555)
Supplement: S4 Table — (PDF) [file pcbi.1014555.s004.pdf]

S4 Table. Myocardial conduction velocities.

**Parameter**	**Description**	**Baseline**	**Range**
CV_f	Myocardial conduction velocity along fiber (in m/s)	0.7	$[0.3, 0.8]$
$\nabla_z CV_f$	Longitudinal gradient for CV_f	1	$[1, 1.67]$
$\nabla_\rho CV_f$	Transmural gradient for CV_f	1	$[0.6, 1]$
$aniso\_ratio$	Anisotropy ratio	0.492	$[0.35, 0.5]$
CV_f^{SE}/CV_f	Scaling factor for CV in SE-layer	1.33	$[1.1, 8.75]$
