# Supplementary material for: Body surface potential driven personalisation of electrophysiological digital twins in hypertrophic cardiomyopathy
Source: PLoS Comput Biol. 2026 Jul 27;22(7):e1014555. doi: 10.1371/journal.pcbi.1014555 (PMC13432148; doi:10.1371/journal.pcbi.1014555)
Supplement: S5 Table — (PDF) [file pcbi.1014555.s005.pdf]

S5 Table. HCM-specific ionic remodelling parameters. Modifications to the ToR-ORd-dynCl baseline model to incorporate HCM-specific electrophysiological remodelling based on experimental measurements of ion channel expression and functions [1] [2]. Values represent percentage changes relative to the baseline ToR-ORd-dynCl model. Positive values indicate increases; negative values indicate reductions.

**Parameter**	**Description**	**Change (%)**
Based on current density		
G_{NaL}	Late sodium current conductance	+165
P_{Ca}	L-type calcium current conductance	+25
G_{to}	Transient outward potassium current conductance	-80
Based on protein expression		
G_{NCX}	Sodium-calcium exchanger conductance	+50
J_{up}	Multiplier of the uptake of calcium into the SR	-35
J_{rel}	Multiplier of the release calcium current from the SR	-30
Based on mRNA expression		
G_{K1}	Inward rectifier potassium current conductance	-30
G_{Kr}	Rapid delayed rectifier potassium current conductance	-35
G_{Ks}	Slow delayed rectifier potassium current conductance	-55
Additional modifications [3] [4]		
P_{NaK}	Sodium-potassium pump conductance	-30
km_{trpn}	Calcium affinity of troponin	-7

References

1. Tomek J, Bueno-Orovio A, Passini E, Zhou X, Minchole A, Britton O, et al. Development, calibration, and validation of a novel human ventricular myocyte model in health, disease, and drug block. eLife. 2019 dec;8:e48890. doi:10.7554/eLife.48890.

2. Coppini R, Ferrantini C, Yao L, Fan P, Lungo MD, Stillitano F, et al. Late Sodium Current Inhibition Reverses Electromechanical Dysfunction in Human Hypertrophic Cardiomyopathy. Circulation. 2013;127(5):575-84. doi:10.1161/CIRCULATIONAHA.112.134932.

3. Passini E, Mincholé A, Coppini R, Cerbai E, Rodriguez B, Severi S, et al. Mechanisms of pro-arrhythmic abnormalities in ventricular repolarisation and anti-arrhythmic therapies in human hypertrophic cardiomyopathy. Journal of molecular and cellular cardiology. 2016;96:72-81. doi:https://doi.org/10.1016/j.yjmcc.2015.09.003.

4. Robinson P, Griffiths PJ, Watkins H, Redwood CS. Dilated and Hypertrophic Cardiomyopathy Mutations in Troponin and -Tropomyosin Have Opposing Effects on the Calcium Affinity of Cardiac Thin Filaments. Circulation Research. 2007;101(12):1266-73. doi:10.1161/CIRCRESAHA.107.156380.
