# Supplementary material for: Body surface potential driven personalisation of electrophysiological digital twins in hypertrophic cardiomyopathy
Source: PLoS Comput Biol. 2026 Jul 27;22(7):e1014555. doi: 10.1371/journal.pcbi.1014555 (PMC13432148; doi:10.1371/journal.pcbi.1014555)
Supplement: S6 Table — (PDF) [file pcbi.1014555.s006.pdf]

S6 Table. Ion channel and transporter genes exhibiting regional expression gradients in human ventricles. Summary of cardiac ion channels, pumps, and exchangers with documented transmural and/or apico-basal expression heterogeneity, along with their corresponding genes and protein subunits. References indicate studies reporting mRNA or protein expression gradients that informed model parametrization.

Current	Gene(s)/Protein(s)	References
I_{Kr} (rapid delayed rectifier)	KCNH2 (hERG/Kv11.1), KCNE2	[1], [2], [3], [4], [5], [6], [7], [8]
I_{Kb} (background K^+)	KCNK1 (TWIK-1), KCNK2 (TREK-1), KCNK3 (TASK-1), KCNK9 (TASK-3)	[9], [10], [11]
I_{NaK} (Na^+/K^+ pump)	ATP1A1/A2/A3 (α subunits), ATP1B1/B2/B3 (β subunits), FXYD1	[11]
I_{NaL} (late Na^+)	SCN5A (Nav1.5), SCN1B/2B/3B/4B (β subunits), CaMKII, Calmodulin	[2], [3], [11], [12]
I_{CaL} (L-type Ca^{2+})	CACNA1C (Cav1.2), CACNB2 (β subunit)	[2], [3], [11]
I_{NCX} (Na^+/Ca^{2+} exchanger)	SLC8A1 (NCX1)	[2], [3], [11], [13], [14]
I_{to} (transient outward K^+)	KCND3 (Kv4.3), KCND2 (Kv4.2), KCNIP2 (KChIP2), DPP6, DPP10	[1], [2], [4], [6], [7], [11], [15]

References

1. Opthof T, Remme CA, Jorge E, Noriega F, Wiegerinck RF, Tasiam A, et al. Cardiac activation-repolarization patterns and ion channel expression mapping in intact isolated normal human hearts. Heart rhythm. 2017;14(2):265-72. doi:https://doi.org/10.1016/j.hrthm.2016.10.010.

2. Bartos DC, Grandi E, Ripplinger CM. Ion channels in the heart. Comprehensive physiology. 2015;5(3):1423-64. doi:https://doi.org/10.1002/j.2040-4603.2015.tb00647.x.

3. Papp R, Bett GC, Lis A, Rasmusson RL, Baczkó I, Varró A, et al. Genomic upregulation of cardiac Cav1. 2α and NCX1 by estrogen in women. Biology of sex Differences. 2017;8(1):26. doi:https://doi.org/10.1186/s13293-017-0148-4.

4. Jeevaratnam K, Chadda KR, Huang CLH, Camm AJ. Cardiac Potassium Channels: Physiological Insights for Targeted Therapy. Journal of Cardiovascular Pharmacology and Therapeutics. 2018;23(2):119-29. PMID: 28946759. doi:10.1177/1074248417729880.

5. Cheng J, Kamiya K, Liu W, Tsuji Y, Toyama J, Kodama I. Heterogeneous distribution of the two components of delayed rectifier K+ current: a potential mechanism of the proarrhythmic effects of methanesulfonanilideclass III agents. Cardiovascular Research. 1999 07;43(1):135-47. doi:10.1016/S0008-6363(99)00061-9.

6. Szentadrassy N, Banyasz T, Biro T, Szabo G, Toth BI, Magyar J, et al. Apico–basal inhomogeneity in distribution of ion channels in canine and human ventricular myocardium. Cardiovascular Research. 2005 03;65(4):851-60. doi:10.1016/j.cardiores.2004.11.022.

7. Schram G, Pourrier M, Melnyk P, Nattel S. Differential Distribution of Cardiac Ion Channel Expression as a Basis for Regional Specialization in Electrical Function. Circulation Research. 2002;90(9):939-50. doi:10.1161/01.RES.0000018627.89528.6F.

8. Grant AO. Cardiac Ion Channels. Circulation: Arrhythmia and Electrophysiology. 2009;2(2):185-94. doi:10.1161/CIRCEP.108.789081.

9. Wiedmann F, Frey N, Schmidt C. Two-Pore-Domain Potassium (K2P-) Channels: Cardiac Expression Patterns and Disease-Specific Remodelling Processes. Cells. 2021;10(11). doi:10.3390/cells10112914.

10. Bechard E, Bride J, Le Guennec JY, Brette F, Demion M. TREK-1 in the heart: Potential physiological and pathophysiological roles. Frontiers in Physiology. 2022;13:1095102. doi:https://doi.org/10.3389/fphys.2022.1095102.

11. Gaborit N, Le Bouter S, Szuts V, Varro A, Escande D, Nattel S, et al. Regional and tissue specific transcript signatures of ion channel genes in the non-diseased human heart. The Journal of physiology. 2007;582(2):675-93. doi:https://doi.org/10.1113/jphysiol.2006.126714.

12. Remme CA, Verkerk AO, Hoogaars W, Aanhaanen W, Scicluna BP, Annink C, et al. The cardiac sodium channel displays differential distribution in the conduction system and transmural heterogeneity in the murine ventricular myocardium. Basic research in cardiology. 2009;104(5):511-22. doi:https://doi.org/10.1007/s00395-009-0012-8.

13. Xiong W, Tian Y, DiSilvestre D, Tomaselli GF. Transmural Heterogeneity of $Na^{+} - Ca^{2+}$ Exchange: Evidence for Differential Expression in Normal and Failing Hearts. Circulation Research. 2005;97(3):207-9. doi:10.1161/01.RES.0000175935.08283.27.

14. Chen G, Yang X, Alber S, Shusterman V, Salama G. Regional genomic regulation of cardiac sodium–calcium exchanger by oestrogen. The Journal of physiology. 2011;589(5):1061-80. doi:https://doi.org/10.1113/jphysiol.2010.203398.

15. Soltysinska E, Olesen SP, Christ T, Wettwer E, Varró A, Grunnet M, et al. Transmural expression of ion channels and transporters in human nondiseased and end-stage failing hearts. Pflügers Archiv-European Journal of Physiology. 2009;459(1):11-23. doi:https://doi.org/10.1007/s00424-009-0718-3.
