# Supplementary material for: Body surface potential driven personalisation of electrophysiological digital twins in hypertrophic cardiomyopathy
Source: PLoS Comput Biol. 2026 Jul 27;22(7):e1014555. doi: 10.1371/journal.pcbi.1014555 (PMC13432148; doi:10.1371/journal.pcbi.1014555)
Supplement: S7 Table — (PDF) [file pcbi.1014555.s007.pdf]

S7 Table. Single-cell ToR-ORd-dynCl model parameters. Baseline ionic model parameter values with HCM-specific modifications [1], and corresponding scaling factors used to implement transmural and apico-basal ion channel gradients.

Parameter	Description	Baseline	Scaling factor
$G_{(Ca)Cl}$	Conductance of the Ca^{2+} sensitive Cl^{-} current	0.2843	2.521041116
G_{Clb}	Conductance of the background Cl^{-} current	0.00198	2
G_{Kr}	Conductance of the rapid delayed K^{+} rectifier current	0.0209	4.66248944
G_{K1}	Conductance of the inward K^{+} rectifier current	0.4894	4.768926983
G_{Kb}	Conductance of the background K^{+} current	0.0189	4.851511657
G_{Ks}	Conductance of the slow delayed K^{+} rectifier current	0.0005	4.422539719
G_{to}	Conductance of the transient outward K^{+} current	0.032	4.914328248
P_{NaK}	Conductance of the $Na^{+}K^{+}$ pump	10.8156	4.869860943
G_{Na}	Conductance of the fast Na^{+} current	11.7802	3.026748126
G_{NaL}	Conductance of the slow (late) Na^{+} current	0.0739	2.560412913
P_{Nab}	Conductance of the background Na^{+} current	1.92e-09	2
G_{NCX}	Conductance of the Na^{+}-Ca^{2+} exchanger	0.0051	2.827974351
P_{Ca}	Conductance of the L-type Ca^{2+} current	1.05e-04	3.769499309
G_{pCa}	Conductance of the sarcolemmal Ca^{2+} pump	0.0005	2
P_{Cab}	Conductance of the background Ca^{2+} current	5.92e-08	2
J_{rel}	Multiplier of the release Ca^{2+} flux from the SR	1.076	2
J_{up}	Multiplier of the uptake of Ca^{2+} into the SR	0.65	2
$I_{NaCa,SS}$	Fraction of the Na^{+}-Ca^{2+} exchangers located in the subspace	0.35	2
$I_{CaL,SS}$	Fraction of the L-type channels located in the subspace	0.8	2
BSR	Max concentration of the SR binding sites in the DS	0.047	2
BSL	Max concentration of the sarcolemmal binding sites in the DS	1.124	2
α_{CaMK}	Phoshorilation rate of Ca^{2+}/CaMK	0.05	2
β_{CaMK}	Dephoshorilation rate of Ca^{2+}/CaMK	0.00068	2
$CaMKo$	Fraction of active Ca^{2+}/CaMK binding sites at equilibrium	0.05	2
$cmdn_{max}$	Max calmodulin concentration	0.05	4.286735631
$csqn_{max}$	Max concentration of calsequestrin	10	2.16233917

Continued on next page

$trpn_{max}$	Max troponin C concentration	0.07	2.602332932
km_{trpn}	Half-saturation constant of troponin C	0.0005	2
τ_{Ca}	Time constant of Ca^{2+} diffusion from the cytoplasm to the DS	0.24	2
τ_{Tr}	Time constant of Ca^{2+} diffusion from the junctional to the network SR	60	2

References

1. Tomek J, Bueno-Orovio A, Passini E, Zhou X, Minchole A, Britton O, et al. Development, calibration, and validation of a novel human ventricular myocyte model in health, disease, and drug block. eLife. 2019 dec;8:e48890. doi:10.7554/eLife.48890.
