# Supplementary material for: Body surface potential driven personalisation of electrophysiological digital twins in hypertrophic cardiomyopathy
Source: PLoS Comput Biol. 2026 Jul 27;22(7):e1014555. doi: 10.1371/journal.pcbi.1014555 (PMC13432148; doi:10.1371/journal.pcbi.1014555)
Supplement: S8 Table — (PDF) [file pcbi.1014555.s008.pdf]

S8 Table. Conductivities for all non-myocardial regions in the torso. Baseline values were assigned based on our previous work [1] and variability for organ conductivities were derived using a scaling factor of 2.

**Region**	**Conductivity (S/m)**	**Scaling factor**
Torso cavity	0.2472	2
Skin	0.117	2
Bones	0.05	2
Kidneys, Pancreas, Liver	0.1667	2
Stomach, Spleen	0.1	2
Lungs	0.0714	2
Blood pools	0.6667	2
Atrial wall (left and right)	0.25	2

References

1. Malik S, Cicci L, Qayyum A, Ghelani R, Chow JJ, Young AA, et al. Semi Automated Pipeline to Create Anatomical Twins and Perform Electrophysiology Simulations for Hypertrophic Cardiomyopathy. In: 2024 Computing in Cardiology Conference (CinC). vol. 51. Computing in Cardiology; 2024. doi:10.22489/cinc.2024.394.
