# Supplementary material for: Body surface potential driven personalisation of electrophysiological digital twins in hypertrophic cardiomyopathy
Source: PLoS Comput Biol. 2026 Jul 27;22(7):e1014555. doi: 10.1371/journal.pcbi.1014555 (PMC13432148; doi:10.1371/journal.pcbi.1014555)
Supplement: S9 Table — (PDF) [file pcbi.1014555.s009.pdf]

S9 Table. ECG-Derived Markers Used for Pacing Analysis. The following ECG-derived markers were analysed to characterize pacing-induced changes in ventricular depolarisation and repolarisation relative to intrinsic rhythm. Unless otherwise stated, lead-specific markers were computed for precordial leads V1-V8, and summary statistics were obtained using the median across leads for each subject.

**Marker**	**Description**	**Unit**
QRS_d	Global duration of ventricular depolarisation, measured from the earliest onset to the latest offset of the QRS complex across all leads.	ms
V_d	Lead-specific ventricular activation time, measured from QRS onset to the time of maximum absolute slope of the QRS complex ($\arg\max\lvert dV/dt\rvert$), representing local arrival of the depolarisation wavefront.	ms
$QRS_{max,center}$	Lead-specific temporal center of the QRS complex, calculated as the amplitude-weighted temporal centroid.	ms
ARI	Lead-specific surrogate for local action potential duration, calculated as the difference between local repolarisation and activation times.	ms
RT	Lead-specific repolarisation timing, measured from QRS onset to the time of maximum absolute slope of the T-wave ($\arg\max\lvert dV/dt\rvert$).	ms
T_{max}	Lead-specific time interval from QRS onset to maximum absolute T-wave amplitude.	ms
T_{on}	Lead-specific time instant marking the beginning of the T-wave, representing the onset of ventricular repolarisation.	ms
T_{off}	Lead-specific time instant marking the end of the T-wave, representing the completion of ventricular repolarisation.	ms
