# Supplementary material for: Body surface potential driven personalisation of electrophysiological digital twins in hypertrophic cardiomyopathy
Source: PLoS Comput Biol. 2026 Jul 27;22(7):e1014555. doi: 10.1371/journal.pcbi.1014555 (PMC13432148; doi:10.1371/journal.pcbi.1014555)
Supplement: S10 Table — (PDF) [file pcbi.1014555.s010.pdf]

S10 Table. Associations Between Continuous Clinical Variables and Calibrated Parameters.

Demographic	Parameter	Pearson (r)	Spearman (ρ)
**Age**	$\nabla_\rho CV_{f,LV}$	r = -0.685<br>p = 0.0048	ρ = -0.668<br>p = 0.0065
	$\nabla_Z G_{Kr,LV}$	r = 0.585<br>p = 0.0218	ρ = 0.604<br>p = 0.0172
	$\nabla_Z CV_{f,LV}$	r = -0.560<br>p = 0.0301	—
	RV_mod_ϕ	—	ρ = -0.532<br>p = 0.0412
**Body Surface Area**	RV_sf_z	r = -0.745<br>p = 0.0015	ρ = -0.905<br>p = 0.0000
	$\nabla_\rho CV_{f,LV}$	r = -0.623<br>p = 0.0131	ρ = -0.621<br>p = 0.0134
	$G_{NaL,RV}$	—	ρ = 0.584<br>p = 0.0224
	CV_f^{SE}/CV_f	r = 0.540<br>p = 0.0376	—
**Body Mass Index**	RV_sf_z	r = -0.539<br>p = 0.0380	ρ = -0.782<br>p = 0.0006
	$\nabla_Z G_{Kr,RV}$	r = 0.629<br>p = 0.0121	—
	$\nabla_\rho CV_{f,LV}$	—	ρ = -0.599<br>p = 0.0184
**LV Maximum Wall Thickness (mm)**	RV_sf_ϕ	r = 0.679<br>p = 0.0054	ρ = 0.678<br>p = 0.0055
	$G_{Kr,LV}$	r = -0.577<br>p = 0.0243	ρ = -0.640<br>p = 0.0102
	$P_{Ca,LV}$	r = -0.613<br>p = 0.0152	ρ = -0.604<br>p = 0.0172
	$trpn_{max}$	r = -0.595<br>p = 0.0192	ρ = -0.548<br>p = 0.0344
	$\nabla_\rho G_{Kr,RV}$	r = -0.529<br>p = 0.0424	—

Continued on next page

**LA Diameter (mm)**	G_{bath}	r = 0.742<br>p = 0.0015	ρ = 0.656<br>p = 0.0079
	$\nabla_Z G_{NCX,LV}$	r = 0.658<br>p = 0.0076	ρ = 0.606<br>p = 0.0167
	RV_sf_z	—	ρ = -0.558<br>p = 0.0308
	$\nabla_\rho G_{NCX,LV}$	r = 0.521<br>p = 0.0464	—
**ESC Risk Score of SCD**	$CV_{f,LV}$	r = 0.628<br>p = 0.0123	ρ = 0.554<br>p = 0.0323
	$trpn_{max}$	—	ρ = -0.608<br>p = 0.0162
	$\nabla_Z P_{Ca,RV}$	r = -0.604<br>p = 0.0172	ρ = -0.585<br>p = 0.0221
	RV_sf_z	r = 0.573<br>p = 0.0256	—
