# Supplementary material for: Body surface potential driven personalisation of electrophysiological digital twins in hypertrophic cardiomyopathy
Source: PLoS Comput Biol. 2026 Jul 27;22(7):e1014555. doi: 10.1371/journal.pcbi.1014555 (PMC13432148; doi:10.1371/journal.pcbi.1014555)
Supplement: S11 Table — (PDF) [file pcbi.1014555.s011.pdf]

S11 Table. Associations Between Categorical Clinical Variables and Calibrated Parameters.

Demographic	Parameter	Mann-Whitney U Statistic
**Gender**	RV_sf_z	2.000<br>p = 0.0036
	$\nabla_\rho CV_{f,LV}$	4.000<br>p = 0.0048
	$G_{NaL,RV}$	48.000<br>p = 0.0109
	CV_f^{SE}/CV_f	47.000<br>p = 0.0176
**History of syncope**	$\nabla_\rho G_{Kr,LV}$	42.000<br>p = 0.0059
**History of NSVT**	$CV_{f,LV}$	4.000<br>p = 0.0080
