# Supplementary material for: Body surface potential driven personalisation of electrophysiological digital twins in hypertrophic cardiomyopathy
Source: PLoS Comput Biol. 2026 Jul 27;22(7):e1014555. doi: 10.1371/journal.pcbi.1014555 (PMC13432148; doi:10.1371/journal.pcbi.1014555)
Supplement: S1 Fig — (PDF) [file pcbi.1014555.s012.pdf]

S1 Fig. Representative examples of CMR-to-CT heart registration. Anterior (left) and anterolateral (right) views of the CMR-derived (white) and CT-derived (red) heart masks for two patients, with corresponding quantitative metrics.

(A)

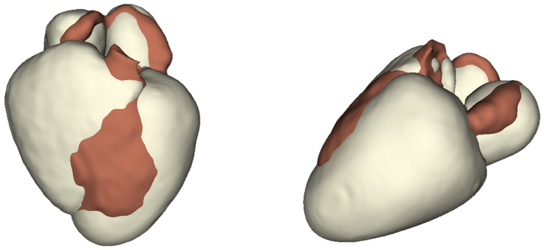

Dice Score	0.86
HD95	7.90 mm
Centroid Offset	1.30 mm
Volume Difference	-16.94 %

(B)

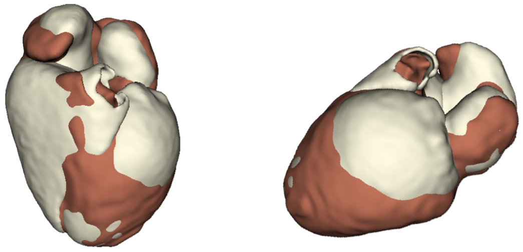

Dice Score	0.85
HD95	11.40 mm
Centroid Offset	4.91 mm
Volume Difference	13.19 %
