# Supplementary material for: Body surface potential driven personalisation of electrophysiological digital twins in hypertrophic cardiomyopathy
Source: PLoS Comput Biol. 2026 Jul 27;22(7):e1014555. doi: 10.1371/journal.pcbi.1014555 (PMC13432148; doi:10.1371/journal.pcbi.1014555)

S2 Fig. Mapping of ECGI vest electrodes to the torso mesh and selection of the reference electrode. (Left) Original 252-electrode geometry from the ECGI vest. (Right) Corresponding electrode locations mapped onto the patient-specific torso surface. A reference electrode (red star) was manually selected at an inferior-anterior left-sided position on the torso. Dashed yellow arrows illustrate correspondence between vest electrodes and their mapped locations on the torso.

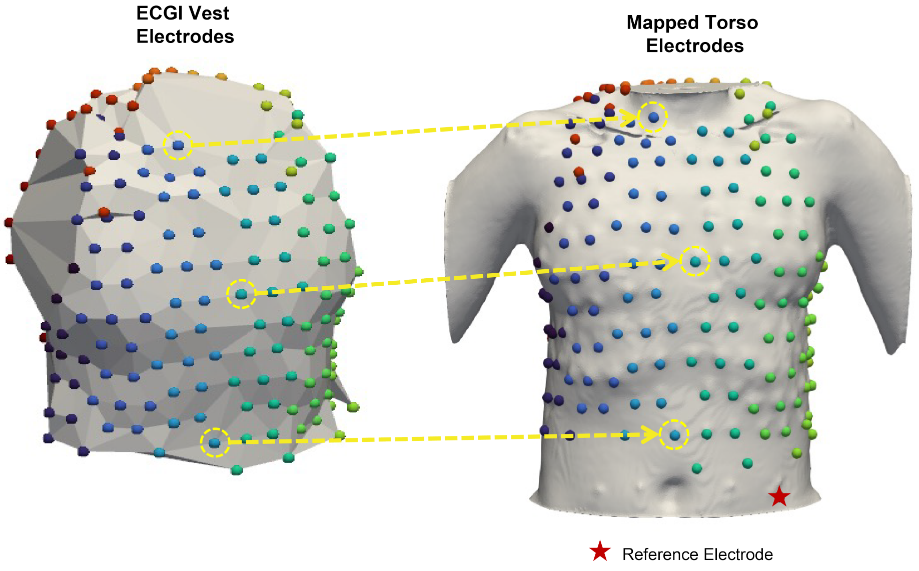

Supplement: S2 Fig — (PDF) [file pcbi.1014555.s013.pdf]
