# Supplementary material for: Body surface potential driven personalisation of electrophysiological digital twins in hypertrophic cardiomyopathy
Source: PLoS Comput Biol. 2026 Jul 27;22(7):e1014555. doi: 10.1371/journal.pcbi.1014555 (PMC13432148; doi:10.1371/journal.pcbi.1014555)

S3 Fig. Fascicular-based modelling of the His-Purkinje system (HPS). (A) Bi-ventricular geometry showing a one-element-thick subendocardial (SE) layer (blue). A zoomed inset highlights the thin SE layer along the endocardial surface. (B) Early activation sites representing HPS root points, positioned within the SE layer. Sites include the left-ventricular septal, posterior, and anterior fascicles (LV_sf, LV_pf, LV_af), and the right-ventricular septal and moderator-band insertions (RV_sf, RV_mod). An anterior view (left) and a septal cross-sectional view (right) illustrate their spatial distribution across both ventricles. RV = right ventricle; LV = left ventricle.

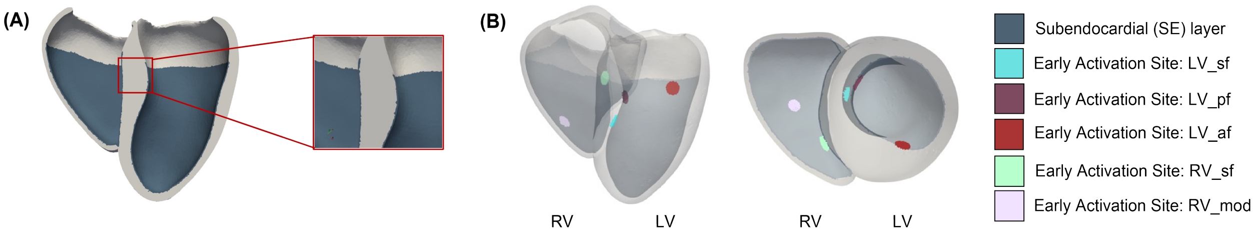

Supplement: S3 Fig — (PDF) [file pcbi.1014555.s014.pdf]
