# Supplementary material for: Body surface potential driven personalisation of electrophysiological digital twins in hypertrophic cardiomyopathy
Source: PLoS Comput Biol. 2026 Jul 27;22(7):e1014555. doi: 10.1371/journal.pcbi.1014555 (PMC13432148; doi:10.1371/journal.pcbi.1014555)

S4 Fig. Global sensitivity analysis of the ToR-ORd-dynCl ionic model. Top: Parameter ranks based on the maximum total-order sensitivity index (S_T) across all outputs. Dashed vertical line denotes the cutoff capturing 90% of the cumulative total sensitivity, separating influential parameters (purple) from low-influence parameters (grey). Bottom: Heatmap of S_T indices showing the contribution of each model parameter (columns) to each output metric (rows). Sensitivity values are normalised across parameters for each output to facilitate comparison of relative influence patterns. The darker the colour, the larger the influence of an input on a particular output. Outputs: Action potential duration (APD), diastolic interval (DI), triangulation (Tri), peak of the action potential (V_{peak}) and resting membrane potential (RMP).

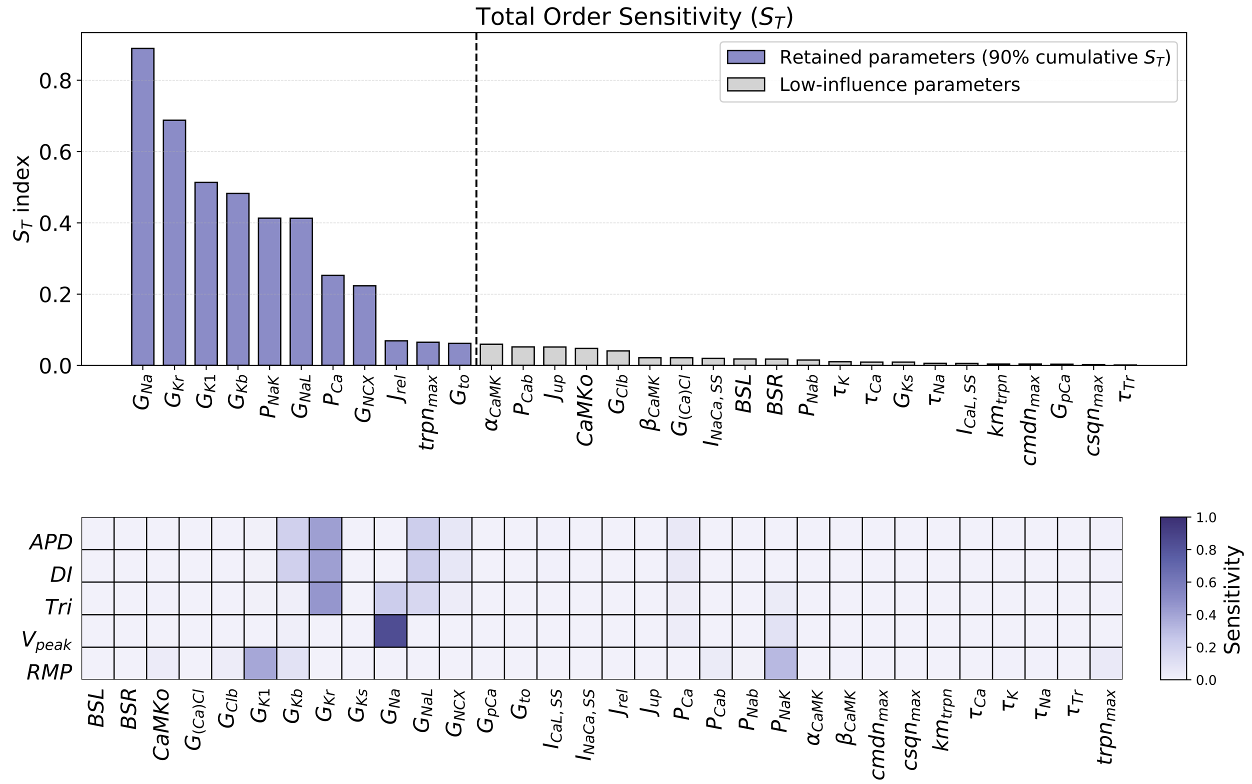

Supplement: S4 Fig — (PDF) [file pcbi.1014555.s015.pdf]
