# Supplementary material for: Body surface potential driven personalisation of electrophysiological digital twins in hypertrophic cardiomyopathy
Source: PLoS Comput Biol. 2026 Jul 27;22(7):e1014555. doi: 10.1371/journal.pcbi.1014555 (PMC13432148; doi:10.1371/journal.pcbi.1014555)

S5 Fig. QRS-complex parameter screening across patients. Heatmap showing the normalised cumulative variance contribution of QRS-related model parameters across patients. Parameters retained within the 90% cumulative variance threshold for each patient are displayed, with rows ordered by cohort-level consistency. Colour intensity reflects the relative contribution of each parameter to the total variance of QRS-derived outputs.

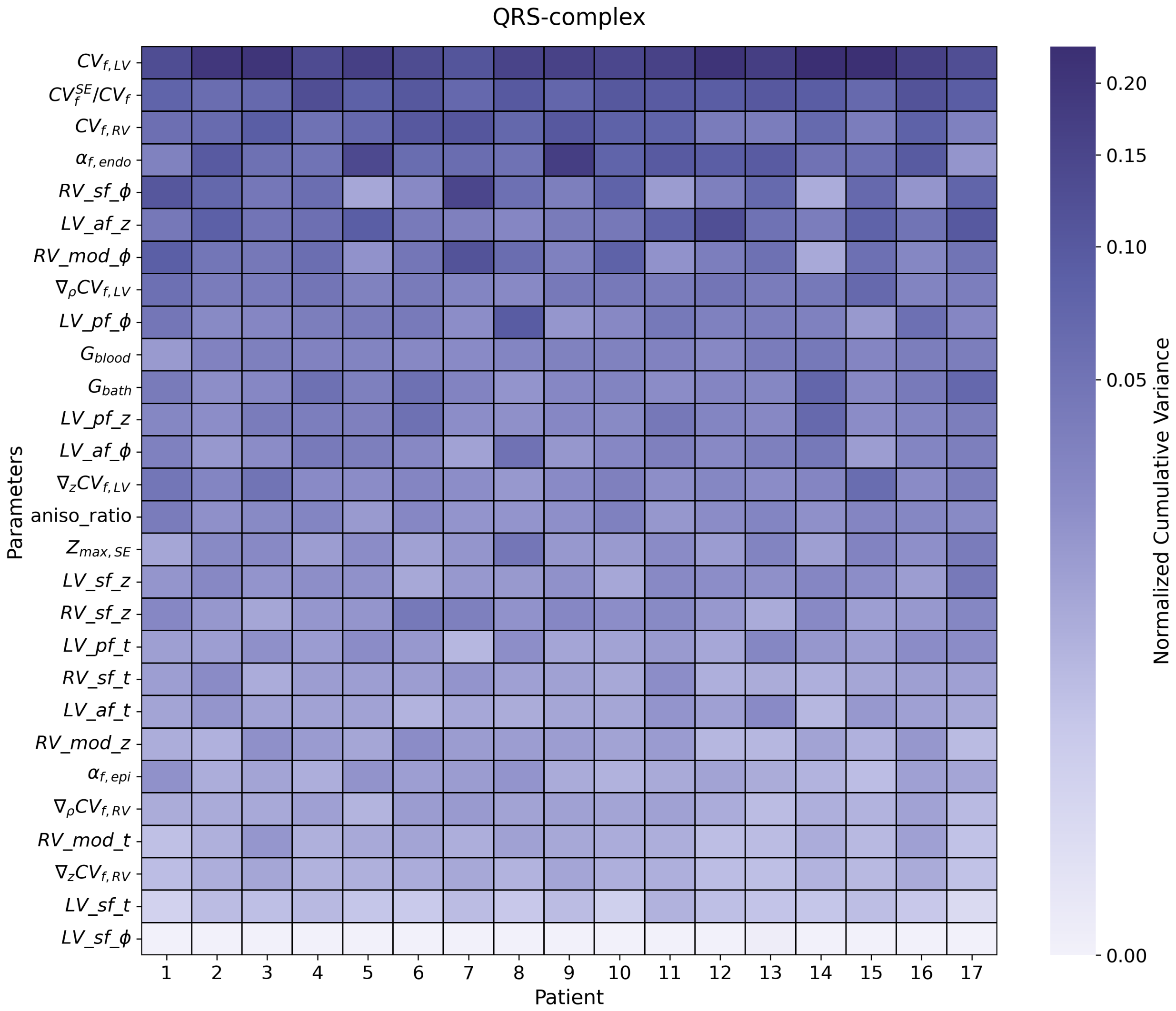

Supplement: S5 Fig — (PDF) [file pcbi.1014555.s016.pdf]
