# Supplementary material for: Body surface potential driven personalisation of electrophysiological digital twins in hypertrophic cardiomyopathy
Source: PLoS Comput Biol. 2026 Jul 27;22(7):e1014555. doi: 10.1371/journal.pcbi.1014555 (PMC13432148; doi:10.1371/journal.pcbi.1014555)

S6 Fig. T-wave parameter screening across patients. Heatmap showing the normalised cumulative variance contribution of T-wave-related model parameters across patients. For each patient, parameters are ranked by their contribution to output variance, and only those retained within the 90% cumulative variance threshold are shown. Colour intensity indicates the relative contribution of each parameter to total variance for a given patient.

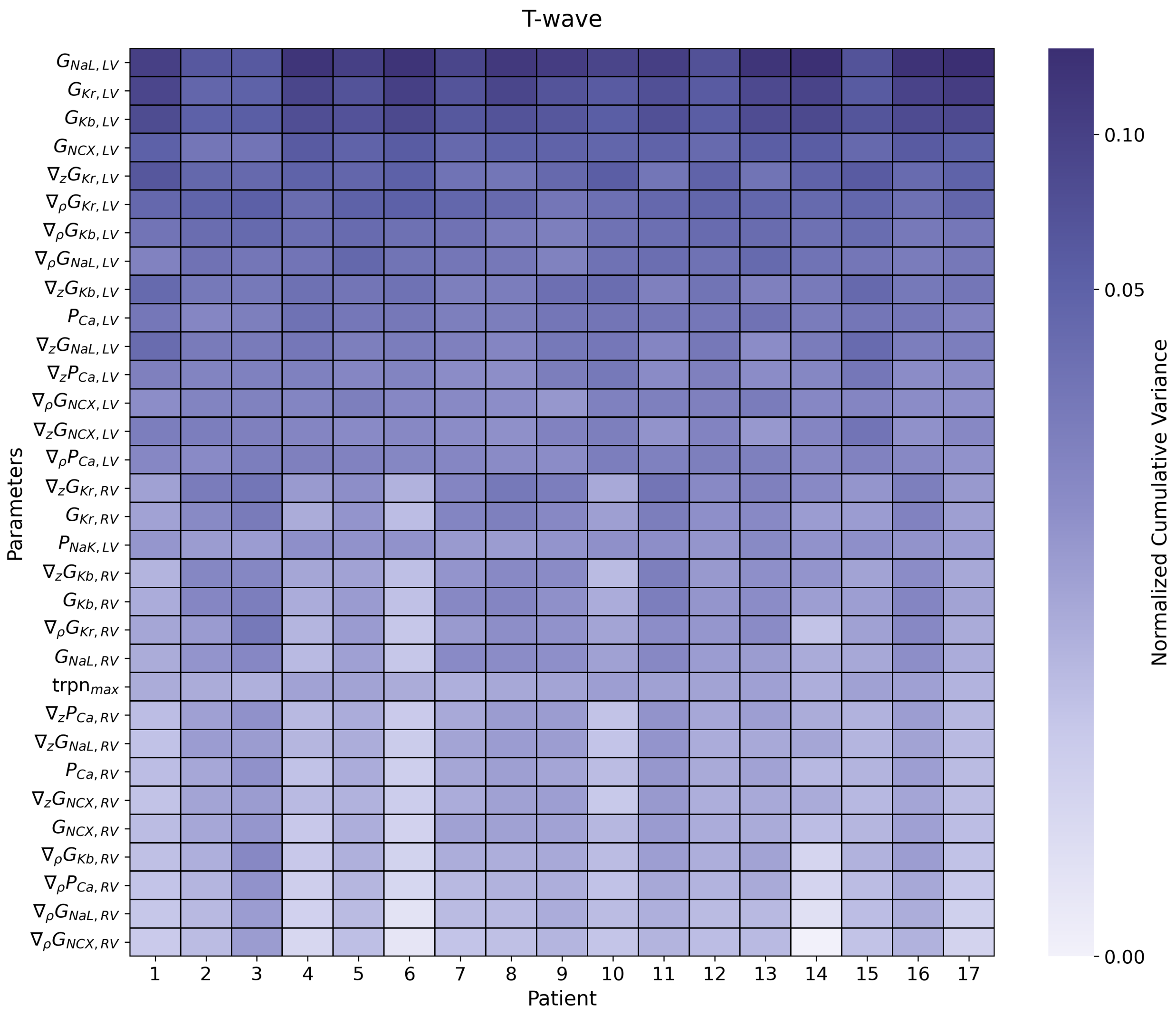

Supplement: S6 Fig — (PDF) [file pcbi.1014555.s017.pdf]
