# Supplementary material for: Body surface potential driven personalisation of electrophysiological digital twins in hypertrophic cardiomyopathy
Source: PLoS Comput Biol. 2026 Jul 27;22(7):e1014555. doi: 10.1371/journal.pcbi.1014555 (PMC13432148; doi:10.1371/journal.pcbi.1014555)

S7 Fig. Emulator-based Bayesian history matching framework. Forward simulations map input model parameters (p) to output signals, which are approximated using emulators. Emulator predictions are compared against clinical ground-truth to compute an implausibility measure, used by history matching to partition the parameter space into implausible (red) and non-implausible (green) regions.

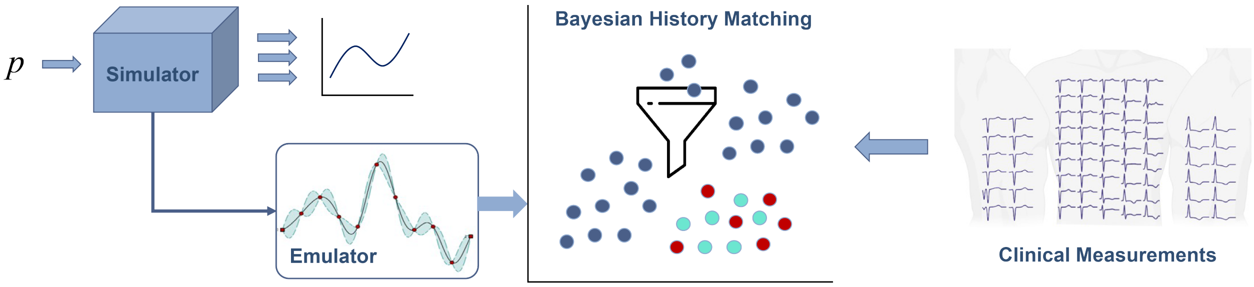

Supplement: S7 Fig — (PDF) [file pcbi.1014555.s018.pdf]
