# Supplementary material for: Body surface potential driven personalisation of electrophysiological digital twins in hypertrophic cardiomyopathy
Source: PLoS Comput Biol. 2026 Jul 27;22(7):e1014555. doi: 10.1371/journal.pcbi.1014555 (PMC13432148; doi:10.1371/journal.pcbi.1014555)

S8 Fig. Calibration computational cost quantified as history matching (HM) iterations or "waves". Stacked bars show the number of HM iterations required for QRS-complex (blue) and T-wave (purple) parameter refinement for each patient.

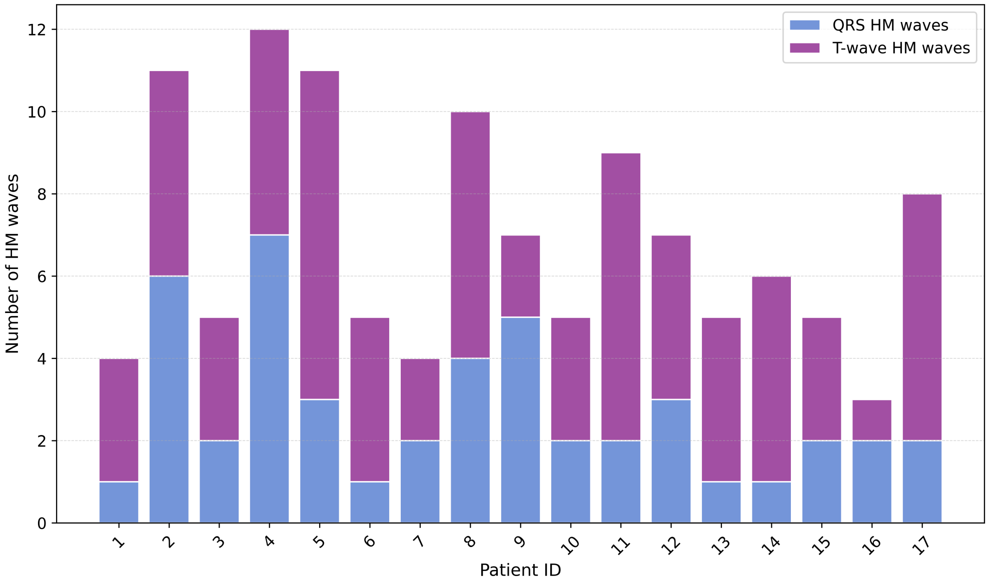

Supplement: S8 Fig — (PDF) [file pcbi.1014555.s019.pdf]
