# Supplementary material for: Body surface potential driven personalisation of electrophysiological digital twins in hypertrophic cardiomyopathy
Source: PLoS Comput Biol. 2026 Jul 27;22(7):e1014555. doi: 10.1371/journal.pcbi.1014555 (PMC13432148; doi:10.1371/journal.pcbi.1014555)

S9 Fig. Consistency of calibration performance between representative and full vest electrode sets. Scatter plot comparing per-patient calibration performance on the 50 representative electrodes (x-axis) and the full 252-electrode configuration (y-axis), quantified as the percentage of electrodes achieving moderate-to-good morphological agreement ($PCC \geq 0.6$). Each point corresponds to one patient. Point colour indicates the signed difference between full-vest and representative-electrode match rates ($\Delta = \text{full} - \text{sampled}$), expressed in percentage points. The dashed grey line denotes identity (equal performance on both electrode sets), while the dashed red line shows the least-squares linear fit. The annotated median Δ summarises the cohort-level consistency.

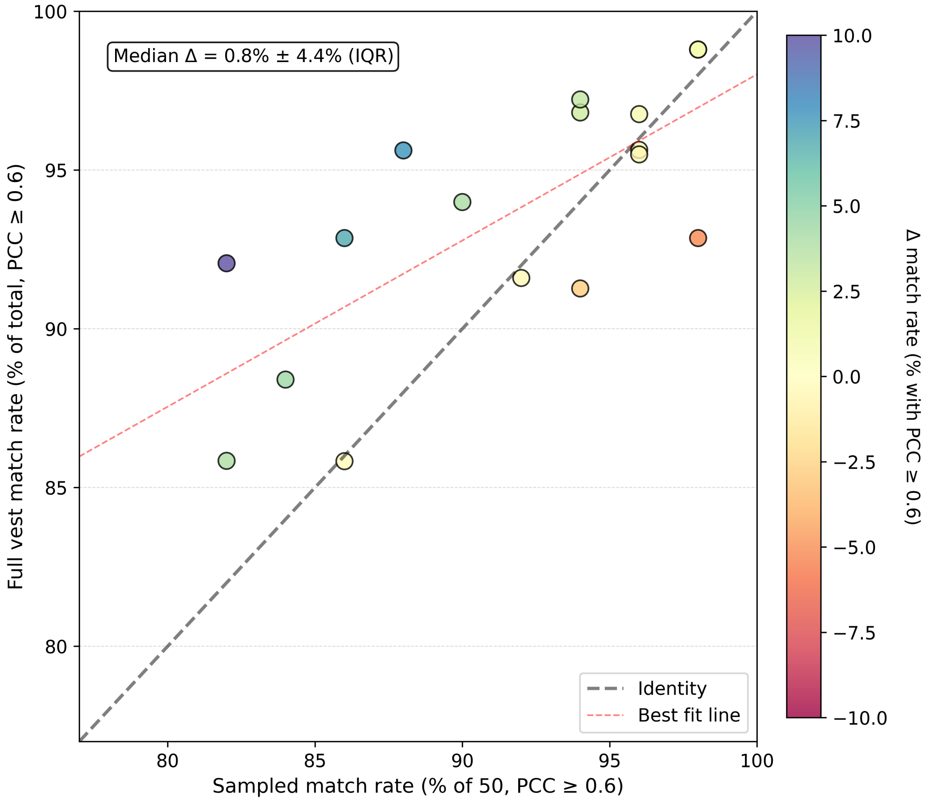

Supplement: S9 Fig — (PDF) [file pcbi.1014555.s020.pdf]
