# Supplementary material for: Body surface potential driven personalisation of electrophysiological digital twins in hypertrophic cardiomyopathy
Source: PLoS Comput Biol. 2026 Jul 27;22(7):e1014555. doi: 10.1371/journal.pcbi.1014555 (PMC13432148; doi:10.1371/journal.pcbi.1014555)

S10 Fig. Spatial distribution of sampled electrodes for representative higher- and lower-performing cases. Anterior and posterior views of the patient-specific torso with corresponding 50 sampled electrodes (black markers) for (A) representative higher-performing patient and (B) representative lower-performing patient. Electrode locations vary patient-to-patient but consistently span both anterior and posterior surfaces.

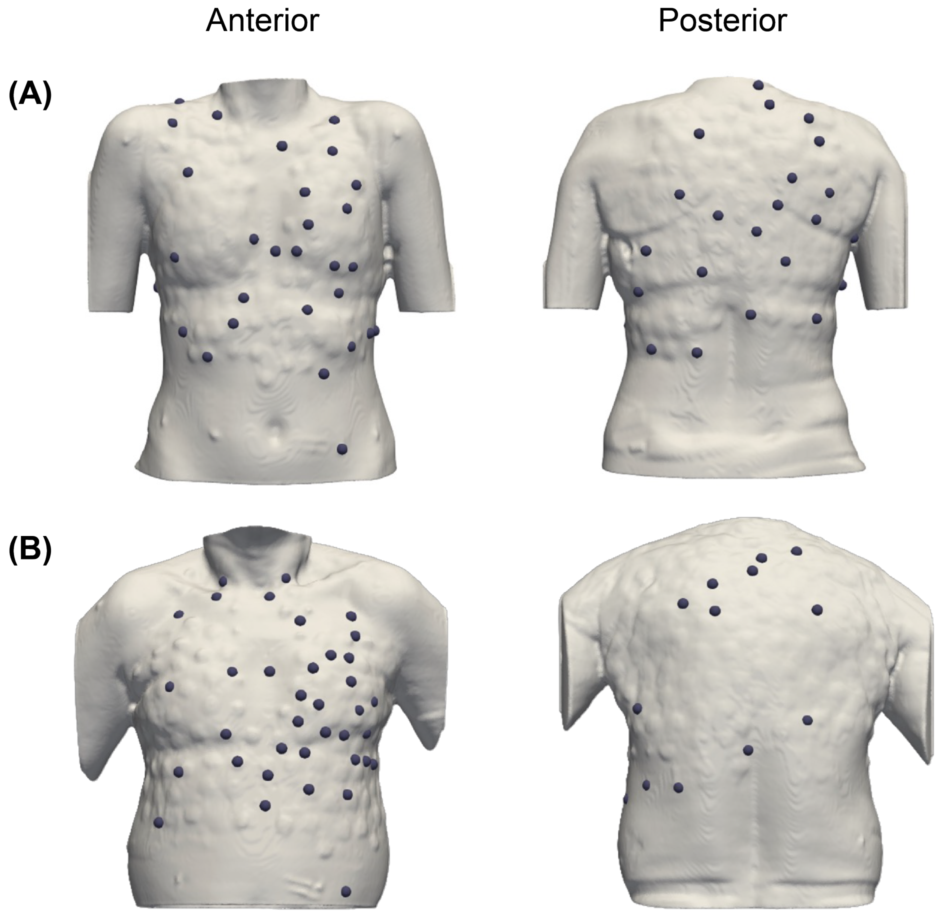

Supplement: S10 Fig — (PDF) [file pcbi.1014555.s021.pdf]
