# Supplementary material for: Body surface potential driven personalisation of electrophysiological digital twins in hypertrophic cardiomyopathy
Source: PLoS Comput Biol. 2026 Jul 27;22(7):e1014555. doi: 10.1371/journal.pcbi.1014555 (PMC13432148; doi:10.1371/journal.pcbi.1014555)

S11 Fig. Association between calibration match percentage and continuous demographic and clinical variables. Scatter plots show the % of sampled electrodes achieving $PCC \geq 0.6$ against (A) age, (B) body mass index (BMI), (C) body surface area (BSA), (D) maximum left-ventricular wall thickness, (E) left atrial diameter, (F) left-ventricular outflow tract (LVOT) gradient, and (G) ESC sudden cardiac death risk score. Red lines indicate least-squares linear fits. Pearson correlation coefficients (r) and associated p-values are shown in each panel.

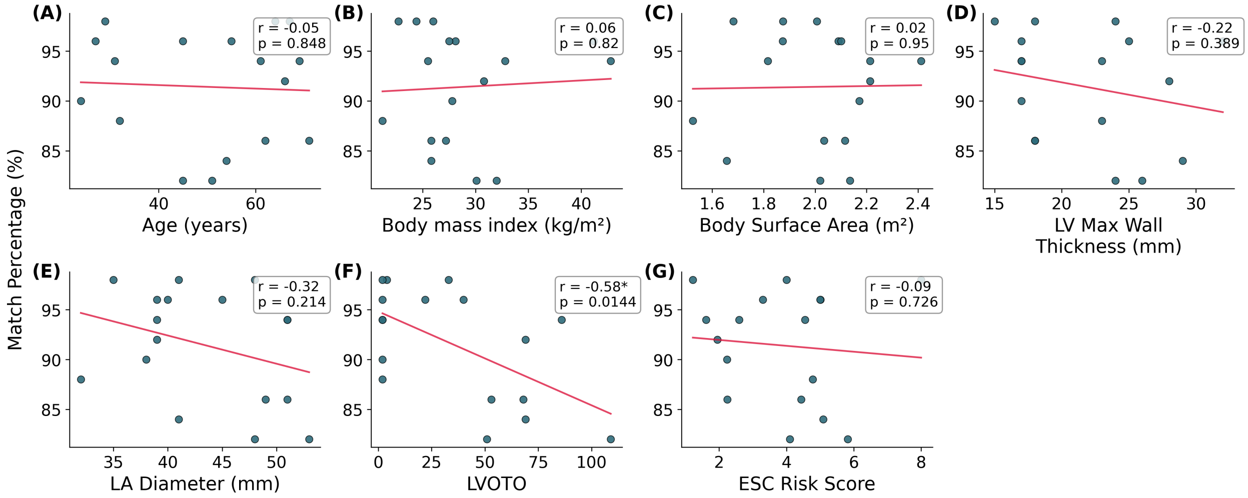

Supplement: S11 Fig — (PDF) [file pcbi.1014555.s022.pdf]
