# Supplementary material for: Body surface potential driven personalisation of electrophysiological digital twins in hypertrophic cardiomyopathy
Source: PLoS Comput Biol. 2026 Jul 27;22(7):e1014555. doi: 10.1371/journal.pcbi.1014555 (PMC13432148; doi:10.1371/journal.pcbi.1014555)

S12 Fig. Calibration match percentage stratified by categorical demographic and clinical variables. % of sampled electrodes achieving $PCC \geq 0.6$ for (A) sex, (B) history of non-sustained ventricular tachycardia (NSVT), and (C) history of syncope. Individual patient values are shown as coloured points, with group means and standard error of the mean indicated in black. Group differences were assessed using Welch's two-sample t-test; corresponding p-values and Cohen's d effect sizes are reported.

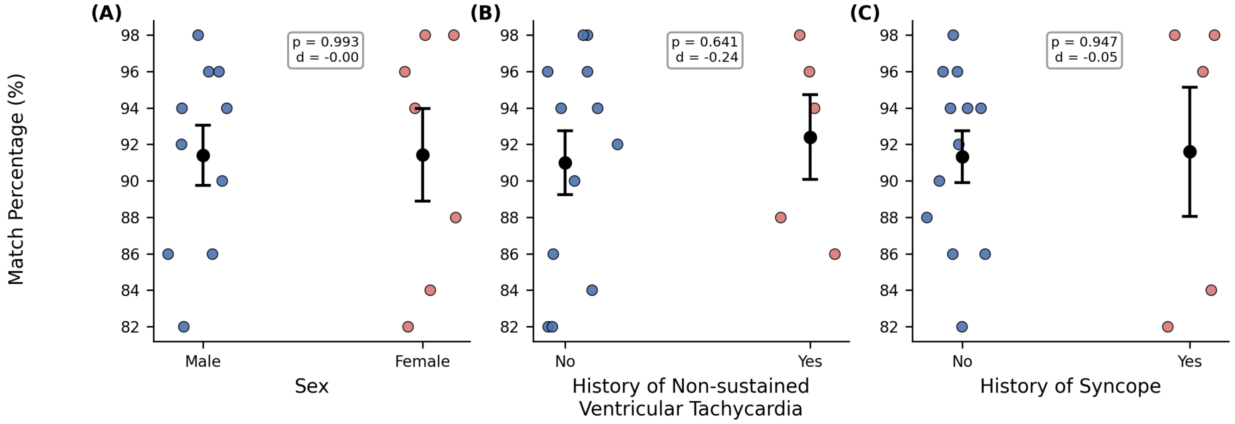

Supplement: S12 Fig — (PDF) [file pcbi.1014555.s023.pdf]
