# Supplementary material for: Body surface potential driven personalisation of electrophysiological digital twins in hypertrophic cardiomyopathy
Source: PLoS Comput Biol. 2026 Jul 27;22(7):e1014555. doi: 10.1371/journal.pcbi.1014555 (PMC13432148; doi:10.1371/journal.pcbi.1014555)

S13 Fig. Physiological distributions of calibrated ventricular conduction parameters. Cohort-level distributions of key conduction parameters inferred by history matching are shown for the best-fit configuration of each patient. Panels summarize baseline left ventricular conduction velocity ($CV_{f,LV}$), apico-basal ($\nabla_Z CV_{f,LV}$) and transmural ($\nabla_\rho CV_{f,LV}$) velocity gradients, subendocardial-to-myocardial conduction scaling (CV_f^{SE}/CV_f), and myocardial anisotropy ratio ($aniso\_ratio$). Boxes indicate interquartile ranges with median values, and points denote individual patients. Red dashed lines denote the bounds of the initial parameter ranges.

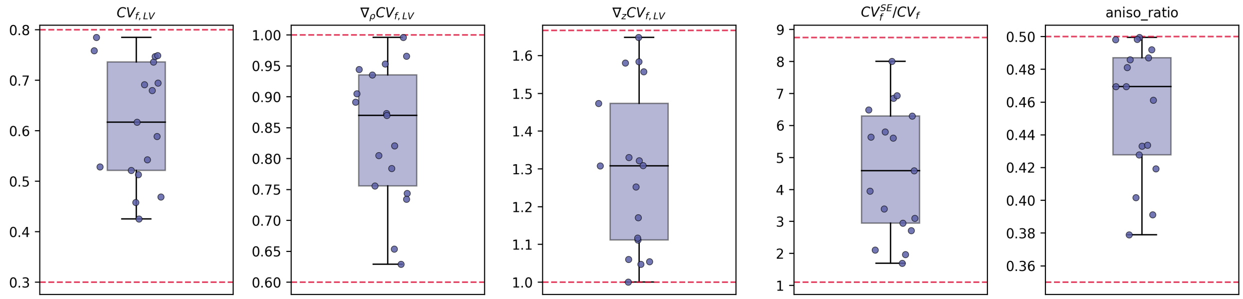

Supplement: S13 Fig — (PDF) [file pcbi.1014555.s024.pdf]
