# Supplementary material for: Body surface potential driven personalisation of electrophysiological digital twins in hypertrophic cardiomyopathy
Source: PLoS Comput Biol. 2026 Jul 27;22(7):e1014555. doi: 10.1371/journal.pcbi.1014555 (PMC13432148; doi:10.1371/journal.pcbi.1014555)

S14 Fig. Distributions of calibrated repolarisation-related ionic parameters. Box-and-scatter plots summarising the cohort-level distributions of all repolarisation-related ionic conductance gradients retained after history matching. Boxes indicate interquartile ranges with median values, and points denote individual patients. Red dashed lines denote prior bounds.

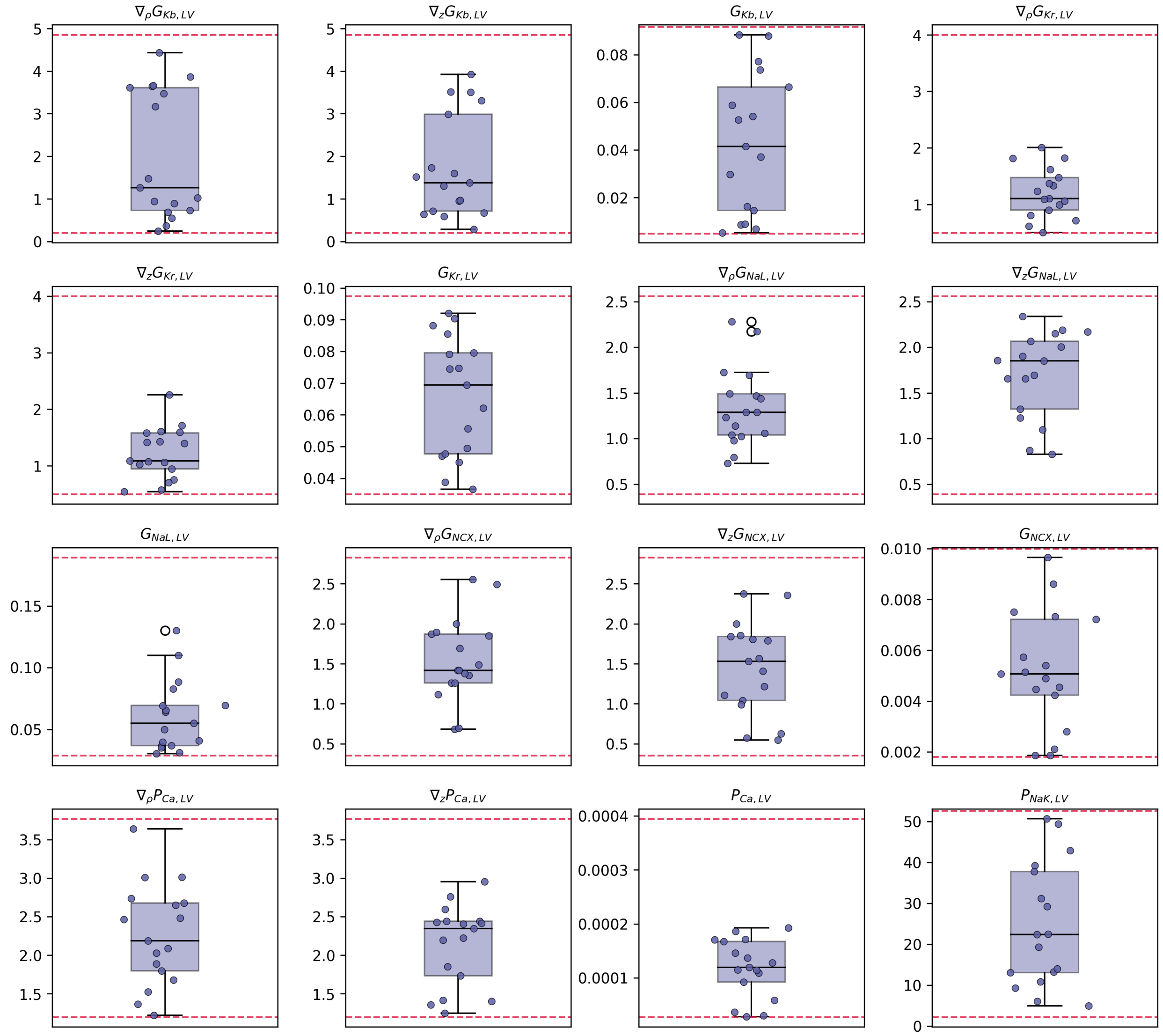

Supplement: S14 Fig — (PDF) [file pcbi.1014555.s025.pdf]
