# Supplementary material for: Body surface potential driven personalisation of electrophysiological digital twins in hypertrophic cardiomyopathy
Source: PLoS Comput Biol. 2026 Jul 27;22(7):e1014555. doi: 10.1371/journal.pcbi.1014555 (PMC13432148; doi:10.1371/journal.pcbi.1014555)

S15 Fig. Exploratory associations between calibrated parameters and categorical clinical phenotypes. Plots show calibrated parameter values stratified by sex (male vs female), history of non-sustained ventricular tachycardia (NSVT), and history of syncope for parameter-phenotype pairs ($p < 0.05$). Individual patient values are shown as coloured points, with group means and standard error of the mean indicated in black. Mann-Whitney U test p-values and corresponding effect sizes (Cohen's d) are reported.

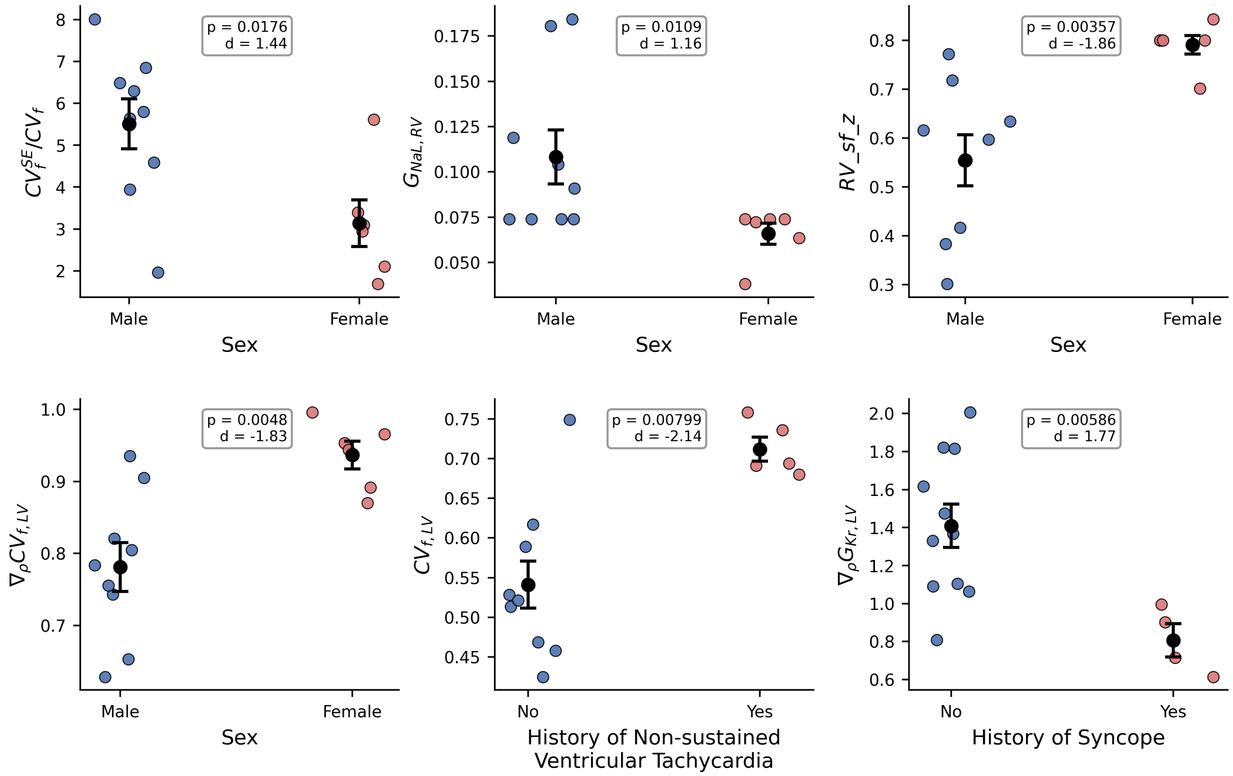

Supplement: S15 Fig — (PDF) [file pcbi.1014555.s026.pdf]
