# Supplementary material for: Body surface potential driven personalisation of electrophysiological digital twins in hypertrophic cardiomyopathy
Source: PLoS Comput Biol. 2026 Jul 27;22(7):e1014555. doi: 10.1371/journal.pcbi.1014555 (PMC13432148; doi:10.1371/journal.pcbi.1014555)

S16 Fig. Exploratory continuous parameter-phenotype associations. Scatter plots show trends between calibrated electrophysiological parameters and continuous clinical demographic for parameter-phenotype pairs ($p < 0.05$). Each point represents an individual patient, solid lines indicate least-squares fits shown for visualization. Corresponding Spearman correlation (ρ) and p-values are reported.

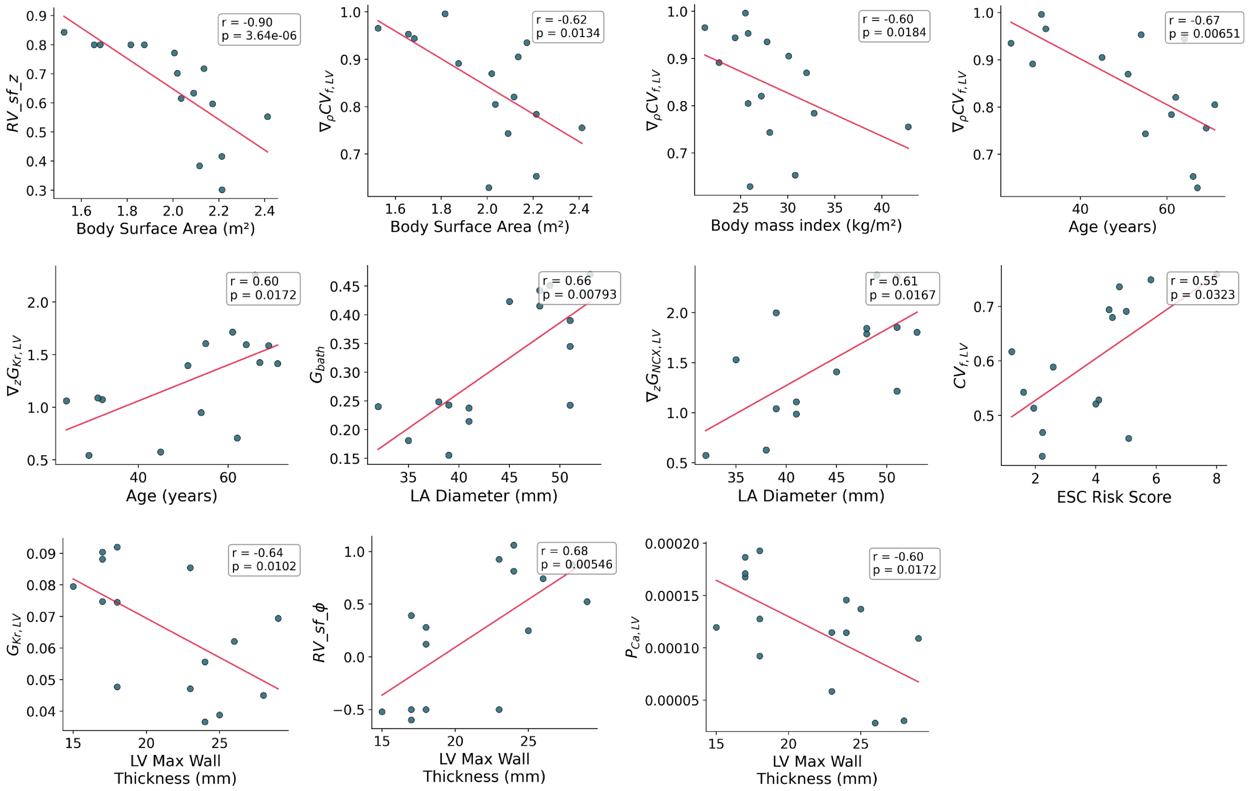

Supplement: S16 Fig — (PDF) [file pcbi.1014555.s027.pdf]
