# Supplementary material for: Body surface potential driven personalisation of electrophysiological digital twins in hypertrophic cardiomyopathy
Source: PLoS Comput Biol. 2026 Jul 27;22(7):e1014555. doi: 10.1371/journal.pcbi.1014555 (PMC13432148; doi:10.1371/journal.pcbi.1014555)
Supplement: S1 Appendix — Detailed descriptions of: (1) medical image segmentation and multimodal registration; (2) the reaction-eikonal framework; (3) the fascicular His-Purkinje system; (4) the framework to assign EP model heterogeneity; (5) scaling factor and estimation of variability ranges; (6) myocardial conduction velocity and conductivities; (7) signal processing; (8) linear-regression-based sensitivity analysis; (9) emulator training; and (10) Bayesian history matching. (PDF) [file pcbi.1014555.s028.pdf]

S1 Appendix

Medical image segmentation and multimodal registration

Segmentation and registration followed our workflow described previously [1]. Non-contrast thoracic CT scans were segmented to create patient-specific torso geometries. Major thoracic and abdominal organs, bones, and skin were segmented automatically using TotalSegmentator [2], an nnU-Net based segmentation algorithm, implemented in 3D Slicer (v5.6.1). Intra-thoracic and intra-abdominal soft tissue, including subcutaneous fat and skeletal muscle, were incorporated into the "torso cavity" segment. Individual segments were smoothed using a Gaussian kernel ($\sigma = 2.5$ mm), followed by joint smoothing to ensure watertight boundaries between adjacent structures.

Cardiac segmentation was performed from long-axis 2D cine CMR slices and short-axis 3D cine CMR volumes using the automated workflow proposed by Qayyum et al [3]. This pipeline utilises a two-stage approach combining self-supervised learning and a hybrid encoder-decoder architecture for slice segmentation with a label-completion U-Net for whole-heart reconstruction. The resulting output included labels for the left/right atria (LA/RA), LV, RV, and the LV myocardium. Regions of hypertrophic myocardium incorrectly assigned to blood pools were manually corrected. Subsequently, the walls of the RV and the LA/RA were added by dilating their corresponding blood pools by 3 mm and 2 mm, respectively, using 3D Slicer to be in agreement with experimental measurements [4] [5] [6] [7]. All cardiac structures were segmented at end-diastole to provide a consistent anatomical reference for mesh generation and EP modelling.

To account for the limited soft tissue resolution of non-contrast CT, whole-heart segmentations from CMR were rigidly registered onto the CT torso geometry. Landmark-based registration was performed in 3D Slicer using manually placed fiducials at the apex, pulmonary valve, and aortic valve across both modalities. A spatial transformation was then computed from the fiducial coordinates using SlicerIGT [8] and applied to align the CMR heart with the CT torso.

Reaction-eikonal framework

Bi-ventricular EP was simulated using the RE formulation [1] [9]. In the RE model, propagation of depolarisation wavefronts is mediated by eikonal-based activation maps that provide wavefront arrival times t_a as a function of location x. The solution t_a is then used to solve the RE model given as:

$$\beta C_m \frac{\partial V_m}{\partial t} = \nabla \cdot \sigma_i \nabla V_m + I_{foot} - \beta I_{ion}$$

where β is the surface-to-volume ratio, C_m is the capacitance per unit area, V_m is the transmembrane voltage, σ_i is the harmonic mean conductivity tensor and I_{ion} is the ionic current. I_{foot} is an additional stimulus current that initiates depolarisation at site x at a time t_a.

Fascicular his-purkinje system

Depolarisation during sinus rhythm is initiated by His-Purkinje mediated activation. As direct measurement of the HPS network topology is not clinically available, it was modelled as a fascicular conduction system [1] [10] comprising five fascicles: left ventricular septal (LV_sf), posterior (LV_pf), and anterior (LV_af) fascicles, right ventricular septal (RV_sf), and right ventricular moderator band (RV_mod). Each was represented by a root point from which the activation wavefront originated. Functional fascicle region was defined as a circular disc of radius 20 μm and thickness equal to 5% of local wall thickness, centred on the root points.

Framework to assign EP model heterogeneity

Previous computational studies [10] [11] have modelled heterogeneity by defining a map based on gradient fields in the UVC space. In this study, we made simplifications by considering heterogeneity to be linear and only along the apico-basal and transmural directions. The transmural gradient was assumed to be constant from apex to base. Baseline parameter values were assigned at the epicardial apex and two gradients: (1) from epicardium to endocardium ($\nabla_\rho X$) and (2) from apex to base ($\nabla_Z X$) were specified. The resulting equations are given by:

$$\theta_X = X_{apex}^{epi} \qquad \nabla_\rho X = \frac{X_{apex}^{endo}}{X_{apex}^{epi}} = \frac{X_{base}^{endo}}{X_{base}^{epi}} \qquad \nabla_Z X = \frac{X_{base}^{epi}}{X_{apex}^{epi}} = \frac{X_{base}^{endo}}{X_{apex}^{endo}}$$

Therefore,

$$\begin{aligned} X_{apex}^{epi} &= \theta_X \\ X_{apex}^{endo} &= \theta_X \nabla_\rho X \\ X_{base}^{epi} &= \theta_X \nabla_Z X \\ X_{base}^{endo} &= \theta_X \nabla_\rho X \nabla_Z X \end{aligned}$$

Scaling factor and estimation of variability ranges

Relative comparisons between regions - right vs left ventricle and epicardium vs endocardium was used to derive variability ranges. Three publicly available datasets of gene expression in healthy human ventricles were selected. Each dataset comprised quantitative measurements of selected genes and included samples from distinct ventricular regions. These were processed as follows:

1. Mean (μ_X) and standard deviation (σ_X) was computed for each gene (X) across samples in each region (r) for a given dataset (d). If available, sex was also taken into account as a category. Scaling factor was then computed using the coefficient of variation (σ_X/μ_X).

   $$C_X^{r,d} = e^{1.96\sqrt{\ln(1+(\sigma_X/\mu_X)^2)}}$$

   Scaling factor translates mRNA information to model parameter ranges.

2. Using $C_X^{r,d}$ for different regions, a median C_X^d was computed and the the maximum value of C across datasets was selected. If multiple genes are responsible for the same ionic current, the maximum value was chosen. This provided a map between genes and model parameters.

3. Finally, variability range was defined by multiplying and dividing the reference value (ref_X) with the scaling factor (C_X) calculated above.

   $$I_X = [ref_X/C_X, ref_X \cdot C_X]$$

C_X defaulted to 2 when no information about the single-cell model parameter was available, implying variability between 50% and 200%.

Myocardial conduction velocity and conductivities

In the RE formulation, CV in the myocardium is specified directly rather than emerging from tissue conductivities. However, extracellular potential (via lead field) computation requires corresponding intra- and extracellular conductivity tensors. Using the strategy proposed by Costa et al. (2013) [12], a lookup table with CV-conductivity mappings was generated. Intermediate values not explicitly listed in the table were interpolated via cubic B-splines and out-of-range values were extrapolated linearly. CV was used as the primary model parameter, as it is physiologically interpretable and more robustly constrained than individual conductivity components.

It is important to note that CV also depends on the chosen ionic model and its parameter values. While the model itself remains fixed, some parameters like the ionic channel and pump densities vary during calibration. Therefore a new CV-conductivity map should be computed for each parameter combination. For simplicity, we have ignored this variation and used a single lookup table generated with reference ToR-ORd-dynCl parameters.

Signal processing

BSPs were filtered using a zero-phase high-pass Butterworth filter with a 0.5 Hz cutoff to remove baseline wander and powerline-interference removal at 50 Hz, consistent with AHA/ACCF/HRS recommendations [13]. Identical cleaning was applied to both clinical and simulated signals, ensuring consistency in the morphological comparison.

The forward EP model simulated single-beat BSPs over one cardiac cycle, whereas clinical BSP recordings captured ten consecutive beats. To enable consistent comparison, clinical BSPs were first segmented into individual beats. Beat segmentation was performed using the NeuroKit2 toolkit [14], which detected R-peaks to identify cardiac cycle boundaries. When automated segmentation failed due to noise or atypical morphology, a fallback procedure was employed that identified simultaneous QRS peaks across multiple electrodes to define single-beat boundaries.

The segmented beats were then used in two distinct stages. For calibration, each beat was projected individually onto the patient-specific PCA basis, and the mean and sample variance of the resulting PC vectors were computed across beats. The mean provided the calibration target, while the across-beat variance was used as part of the HM implausibility metric. Signal features exhibiting greater variability across beats had a correspondingly wider calibration tolerance. For internal validation, where waveform-level morphological agreement was quantified using the PCC, the segmented beats were instead averaged into a single representative beat per electrode prior to comparison with the simulated waveform. Averaging suppressed beat-to-beat fluctuations and provided a stable representative morphology for this purpose.

Linear regression based sensitivity analysis

In the context of sensitivity analysis, a fitted linear model is used to understand how changes in input variables (X_i) affect the response output (Y), using standardized regression coefficients as measures of sensitivity. For one-at-a-time sensitivity analysis, each parameter was perturbed to its upper and lower bounds while keeping others fixed and the influence of parameter X_i on BSP morphology was quantified. Here, the response variable Y are the PC-scores derived from the concatenated BSP signal projected onto PCA basis. Sensitivity index is then defined as:

$$S_i = \frac{\sum_{\ell=1}^{N_{PCA}} (\alpha_i^{(\ell)} |\mathcal{I}_i|)^2}{\sum_{j=1}^{N} \sum_{\ell'=1}^{N_{PCA}} (\alpha_j^{(\ell')} |\mathcal{I}_j|)^2}$$

where $\mathcal{I}_i$ is the variability range of X_i and α_i is the linear regression coefficients obtained for the ℓ-th PC-score. This equation accounts for the uncertainty over the set of values that X_i can assume, thus assigning higher values to inputs with greater uncertainty. Input parameters are ranked from the most to the least influential according to S_i.

Emulator training

Model outputs (y) can be expressed as a function of input parameters (x), such that,

$$y = f(x) \in \mathbb{R} \quad \text{where} \quad x = (x_1, x_2 ... x_N) \in \mathbb{R}^N$$

Emulators provide an efficient mapping between x and $f(x)$, by constructing a statistical model of f and assigning a probability distribution over simulator outputs. The GPE was defined as the sum of a deterministic mean function $h(x)$ and a Gaussian process $g(x)$, given by,

$$h(x) = \beta_0 + \sum_{i=1}^{N} \beta_i x_i \qquad\qquad g(x) \sim GP(0, k(x, x'))$$

where β_i are weights for input parameters and $k(x, x')$ is the covariance kernel function. Considering non-smoothness in the high-dimensional parameter space, a Matern-5/2 kernel was used. Model hyperparameters were optimised using the ADAM optimiser by maximising the Gaussian log-likelihood.

GPE performance was evaluated using the coefficient of determination R^2, measuring goodness of fit, and the independent standard error (ISE) assessing prediction uncertainty calibration. These are calculated as,

$$R^2 = 1 - \frac{SS_{res}}{SS_{tot}}$$

where SS_{res} is the sum of squares of the residual errors and SS_{tot} is the total sum of the errors, and,

$$ISE := \frac{100}{n} \bullet \sum_{i=1}^{n} \left(\frac{|\, y_i^{true} - y_i^{mean}|}{\sqrt{y_i^{\text{var}}}} < 2 \right)$$

where y_i^{true} is the true outputs, and y_i^{mean} and y_i^{var} are the predicted posterior mean and variance of emulator outputs. The Boolean result inside the parentheses is encoded with either 0 (false) or 1 (true). ISE represents the percentage of test predictions within two standard deviations of the true values, providing a measure of uncertainty quantification quality.

Bayesian history matching

HM was performed in sequential 'waves' or iterations. In the initial wave, the full retained parameter space defined the not-ruled-out-yet (NROY) region. A Latin hypercube design (N = $30 \times n_{param}$ samples, where n_{param} is the number of inputs being calibrated) was used to sample this region. Forward simulations were performed for each sample, and GPEs were trained to emulate the resulting PCs. The emulators were then evaluated on a large set of candidate parameter vectors sampled from the current NROY space.

For each candidate vector, an implausibility measure was computed by comparing emulator predictions with the target clinical BSP. The implausibility $I(x)$ for a candidate vector x was defined as:

$$I(x) = \max_{q=1,2...,N_{PCA}} I_q(x) = \max_{q=1,2...,N_{PCA}} \frac{|E[f_q(x)|D] - \mu_q|}{\sqrt{\sigma^2[f_q(x)|D] + \sigma_q^2}}$$

where f_q is the GPE corresponding to the q-th PC score, μ_q is the target value and σ_q is the standard deviation. Candidate vectors with $I(x)$ exceeding a predefined threshold were deemed implausible and discarded. The remaining non-implausible samples defined a reduced NROY region for the next wave. This refined region was sampled (N = $10 \times n_{param}$ samples) for additional forward simulations, and emulators were retrained with the augmented dataset, improving accuracy in regions of interest. HM was repeated with progressively stricter thresholds, targeting a final threshold of 3 [15] or until the NROY space stabilized (i.e., further iterations yielded negligible reduction).

References

1. Malik S, Cicci L, Qayyum A, Ghelani R, Chow JJ, Young AA, et al. Semi Automated Pipeline to Create Anatomical Twins and Perform Electrophysiology Simulations for Hypertrophic Cardiomyopathy. In: 2024 Computing in Cardiology Conference (CinC). vol. 51. Computing in Cardiology; 2024. doi:10.22489/cinc.2024.394.

2. Wasserthal J, Breit HC, Meyer MT, Pradella M, Hinck D, Sauter AW, et al. TotalSegmentator: Robust Segmentation of 104 Anatomic Structures in CT Images. Radiology: Artificial Intelligence. 2023 Sep;5(5). doi:10.1148/ryai.230024.

3. Qayyum A, Xu H, Halliday BP, Rodero C, Lanyon CW, Wilkinson RD, et al.. Transforming Heart Chamber Imaging: Self-Supervised Learning for Whole Heart Reconstruction and Segmentation; 2024. arXiv:2406.06643. doi:https://doi.org/10.48550/arXiv.2406.06643.

4. Ho S, Nihoyannopoulos P. Anatomy, echocardiography, and normal right ventricular dimensions. Heart. 2006;92(suppl 1):i2-i13. doi:https://doi.org/10.1136/hrt.2005.077875.

5. Matsukubo H, Matsuura T, Endo N, Asayama J, Watanabe T. Echocardiographic measurement of right ventricular wall thickness. A new application of subxiphoid echocardiography. Circulation. 1977;56(2):278-84. doi:https://doi.org/10.1161/01.CIR.56.2.278.

6. Li Z, Xu L, et al. Left atrial wall thickness: anatomic aspects relevant to catheter ablation of atrial fibrillation. Chinese Medical Journal. 2012;125(1):12-5. doi:10.3760/cma.j.issn.0366-6999.2012.01.003.

7. Varela M, Morgan R, Theron A, Dillon-Murphy D, Chubb H, Whitaker J, et al. Novel MRI technique enables non-invasive measurement of atrial wall thickness. IEEE transactions on medical imaging. 2017;36(8):1607-14. doi:https://doi.org/10.1109/TMI.2017.2671839.

8. Ungi T, Lasso A, Fichtinger G. Open-source platforms for navigated image-guided interventions. Medical Image Analysis. 2016;33:181-6. doi:https://doi.org/10.1016/j.media.2016.06.011.

9. Neic A, Campos FO, Prassl AJ, Niederer SA, Bishop MJ, Vigmond EJ, et al. Efficient computation of electrograms and ECGs in human whole heart simulations using a reaction-eikonal model. Journal of Computational Physics. 2017;346:191-211. doi:https://doi.org/10.1016/j.jcp.2017.06.020.

10. Gillette K, Gsell MAF, Prassl AJ, Karabelas E, Reiter U, Reiter G, et al. A Framework for the generation of digital twins of cardiac electrophysiology from clinical 12-leads ECGs. Medical Image Analysis. 2021;71:102080. doi:https://doi.org/10.1016/j.media.2021.102080.

11. Camps J, Wang ZJ, Doste R, Berg LA, Holmes M, Lawson B, et al. Harnessing 12-lead ECG and MRI data to personalise repolarisation profiles in cardiac digital twin models for enhanced virtual drug testing. Medical Image Analysis. 2025;100:103361. doi:https://doi.org/10.1016/j.media.2024.103361.

12. Costa CM, Hoetzl E, Rocha BM, Prassl AJ, Plank G. Automatic parameterization strategy for cardiac electrophysiology simulations. In: Computing in Cardiology 2013. IEEE; 2013. p. 373-6. Available from: `https://ieeexplore.ieee.org/abstract/document/6713391/`.

13. Kligfield P, Gettes LS, Bailey JJ, Childers R, Deal BJ, Hancock EW, et al. Recommendations for the standardization and interpretation of the electrocardiogram: part I: the electrocardiogram and its technology: a scientific statement from the American Heart Association Electrocardiography and Arrhythmias Committee, Council on Clinical Cardiology; the American College of Cardiology Foundation; and the Heart Rhythm Society endorsed by the International Society for Computerized Electrocardiology. Circulation. 2007;115(10):1306-24. doi:https://doi.org/10.1161/CIRCULATIONAHA.106.180200.

14. Makowski D, Pham T, Lau ZJ, Brammer JC, Lespinasse F, Pham H, et al. NeuroKit2: A Python toolbox for neurophysiological signal processing. Behavior Research Methods. 2021 feb;53(4):1689-96. doi:10.3758/s13428-020-01516-y.

15. Pukelsheim F. The Three Sigma Rule. The American Statistician. 1994;48(2):88-91. doi:10.1080/00031305.1994.10476030.
